# Supplementary material for: Distinct Compartmentalization of the Chemokines CXCL1 and CXCL2 and the Atypical Receptor ACKR1 Determine Discrete Stages of Neutrophil Diapedesis
Source: Immunity. 2018 Dec 18;49(6):1062–1076.e6. doi: 10.1016/j.immuni.2018.09.018 (PMC6303217; doi:10.1016/j.immuni.2018.09.018)
Supplement: Document S1. Figures S1–S6 [file mmc1.pdf]

Immunity, Volume *49*

Supplemental Information

Distinct Compartmentalization of the Chemokines CXCL1 and CXCL2 and the Atypical Receptor ACKR1 Determine Discrete Stages of Neutrophil Diapedesis

Tamara Girbl, Tchern Lenn, Lorena Perez, Loïc Rolas, Anna Barkaway, Aude Thiriot, Carlos del Fresno, Eleanor Lynam, Elin Hub, Marcus Thelen, Gerard Graham, Ronen Alon, David Sancho, Ulrich H. von Andrian, Mathieu-Benoit Voisin, Antal Rot, and Sussan Nourshargh

Supplemental Information

Supplemental Figures

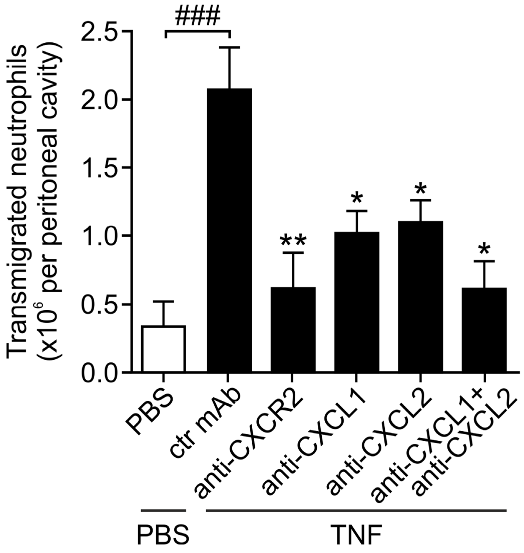

Figure S1 (related to Figure 1): TNF-induced neutrophil migration into the peritoneal cavity is dependent on both CXCL1 and CXCL2. Neutrophil extravasation in the peritoneal cavity of WT mice after intraperitoneal (i.p.) administration of PBS or TNF (300 ng, 4 h). Control (ctr) or blocking mAbs against CXCR2, CXCL1, CXCL2 or CXCL1 + CXCL2 were injected intravenously (i.v.) 10 min prior to TNF (n = 3 -14 mice per group, 9 independent experiments). Means ± SEM, *$p<0.05$, **$p<0.01$ as compared to TNF + ctr mAb treatment and ###$p< 0.001$ as indicated.

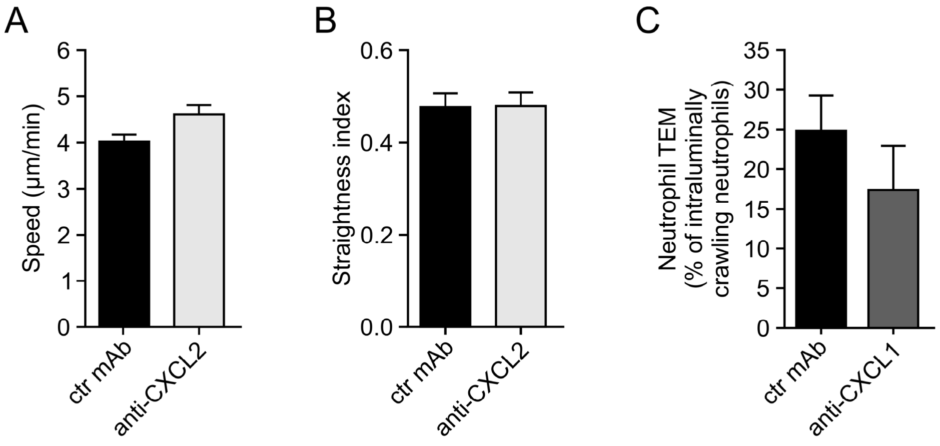

Figure S2 (related to Figure 2): Intraluminal neutrophil crawling dynamics and transendothelial cell migration (TEM) in TNF-stimulated cremaster muscles. (A-B) Intraluminal neutrophil crawling speed (A) and straightness (straightness index = displacement/length of migratory path, B) in TNF-stimulated cremaster muscles of *Lyz2-EGFP-ki;Acta2-RFPcherry-Tg* mice injected i.v. with ctr or blocking anti-CXCL2 mAbs (10 min prior to TNF) as determined by confocal IVM (n = 6 mice per group) from 12 independent experiments. (C) Percentages of intraluminally crawling neutrophils that exhibited TEM in TNF-treated cremaster muscles post treatment with i.s. ctr or anti-CXCL1 mAb (n = 5-10 mice per group) involving 15 independent experiments. Means ± SEM.

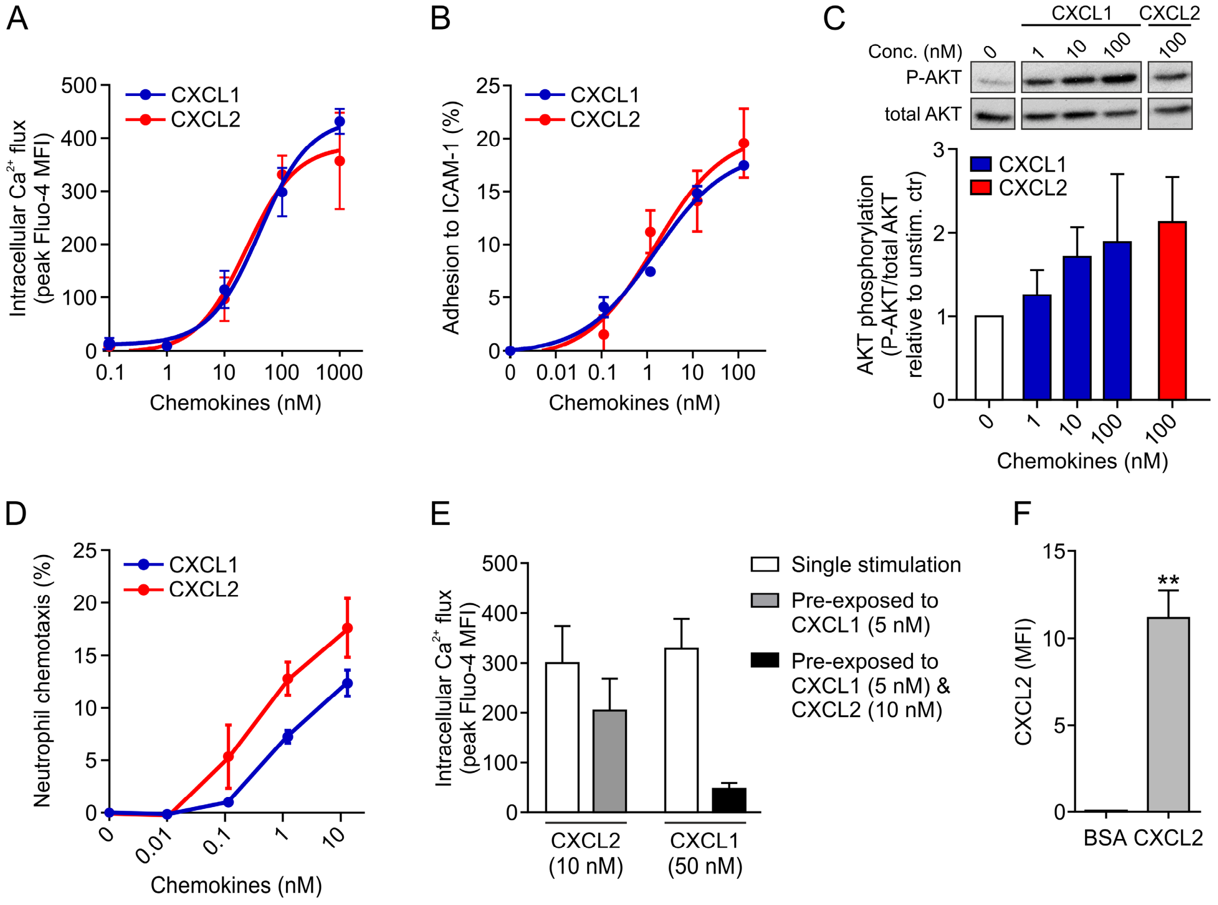

Figure S3 (related to Figure 4): CXCL1 and CXCL2 exhibit similar potencies in inducing neutrophil responses *in vitro*. (A) Intracellular Ca^{2+} flux in isolated bone marrow neutrophils loaded with the calcium indicator Fluo-4 and stimulated with CXCL1 or CXCL2. Peak Fluo-4 mean fluorescence intensities (MFIs) within 2 min after chemokine stimulation minus baseline MFIs, as determined by flow cytometry from 3 independent experiments are shown (n = 3). (B) Neutrophil adhesion to ICAM-1-coated plates as induced by CXCL1 or CXCL2 (n = 2) from 2 independent experiments. (C) Representative Western blot analysis of phospho-AKT (P-AKT) and total AKT and quantification of P-AKT in neutrophils stimulated with CXCL1 or CXCL2 (n = 6) from 4 independent experiments. (D) Neutrophil chemotaxis in response to CXCL1 or CXCL2 as determined by Transwell chemotaxis assays (n = 4) from 4 independent experiments. (E) Quantification of intracellular Ca^{2+} flux in neutrophils after single or serial stimulation with CXCL1 and CXCL2 as expressed as peak Fluo-4 MFIs (n = 4) from 4 independent experiments. (F) MFI of CXCL2 immunostaining on Transwell filters coated with BSA or CXCL2 (n = 3) involving 3 independent experiments. Means ± SEM, **p<0.01.

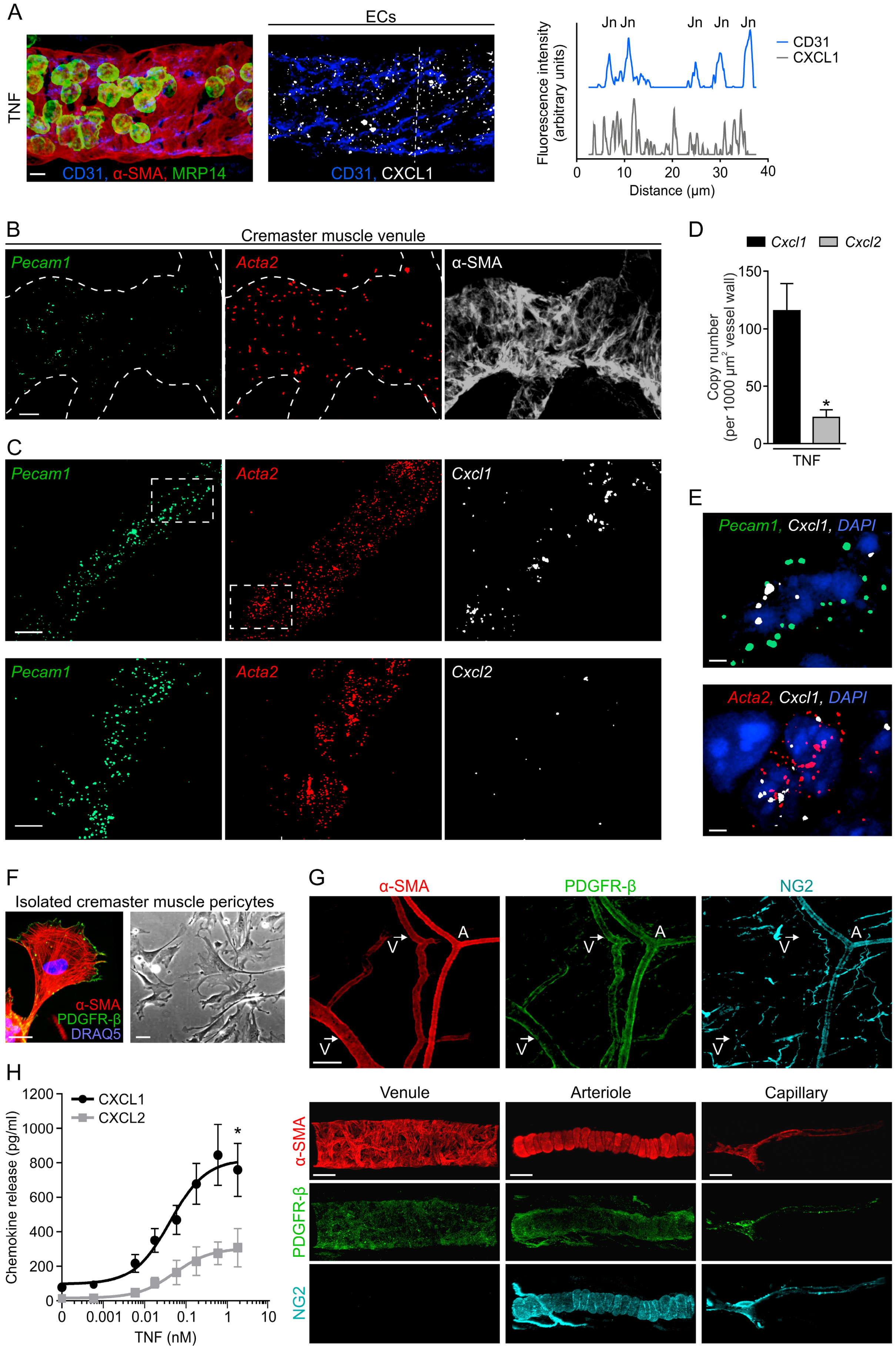

Figure S4 (related to Figure 5): Cremaster muscle ECs and pericytes are an abundant source of CXCL1. (A) TNF-stimulated cremaster muscles were immunostained for CXCL1,

CD31 (ECs), α-SMA (pericytes) and MRP14 (neutrophils) (left panel shows a representative confocal image). Representative confocal image illustrating CXCL1 staining within an EC isosurface mask (middle) and CD31 and CXCL1 immunofluorescence (IF) intensities along the dashed line cutting across 5 EC junctions (Jn, right) are shown. Images and line intensity graphs are representative of 4 independent experiments. Scale bar, 5 µm. (B-E) RNA fluorescent *in situ* hybridization (FISH) of TNF-stimulated cremaster muscles. (B) FISH was validated for the analysis of mRNAs in venular walls by robust detection of *Pecam1* and *Acta2* mRNAs (established markers for EC and pericytes, respectively), in venules concomitantly IF stained for α-SMA and analyzed by confocal microscopy. (C) Confocal images showing hybridization signals for *Pecam1*, *Acta2* and *Cxcl1* (top) or *Cxcl2* (bottom). Images in B and C are representative of 3 independent experiments. Scale bars, 10 µm. (D) Quantification of *Cxcl1* and *Cxcl2* mRNA copies in vascular walls (n = 3-6 mice per group) from 3 independent experiments. (E) Enlarged images of boxed regions in Panel C, showing *Cxcl1* and *Pecam1* in an EC (top) and *Cxcl1* and *Acta2* in a pericyte (bottom). Cell nuclei were stained with DAPI. Scale bars, 2 µm. (F-H) Isolation and analysis of murine cremaster muscle pericytes. (F) Isolated pericytes exhibited a venular pericyte expression profile (α-SMA^+PDGFR-$\beta^+$$NG2^-$) as determined by IF staining and confocal microscopy (left). Lack of expression of NG2 is not shown for clarity. The cells also displayed a stellate morphology that is typical for pericytes, as shown by phase contrast microscopy (right). Images are representative of 3 independent experiments. Scale bars, 30 µm. (G) The RFP^+PDGFR-$\beta^+$$NG2^-$ molecular signature uniquely distinguishes venular wall pericytes from arteriolar and capillary smooth muscle cells and pericytes *in vivo*, as shown by IF staining and confocal microscopy of cremaster muscles of WT mice. V, venule; A, arteriole. Scale bars, 100 µm (top) and 20 µm (bottom). The images are representative of 2 independent experiments. (H) CXCL1 and CXCL2 levels in the cell culture medium of isolated primary α-SMA^+PDGFR-$\beta^+$$NG2^-$ cremaster muscle pericytes stimulated with TNF for 4 h as quantified by ELISA (n = 3) from 3 independent experiments. Means ± SEM, *$p<0.05$ (maximal CXCL1 and CXCL2 release was compared in H).

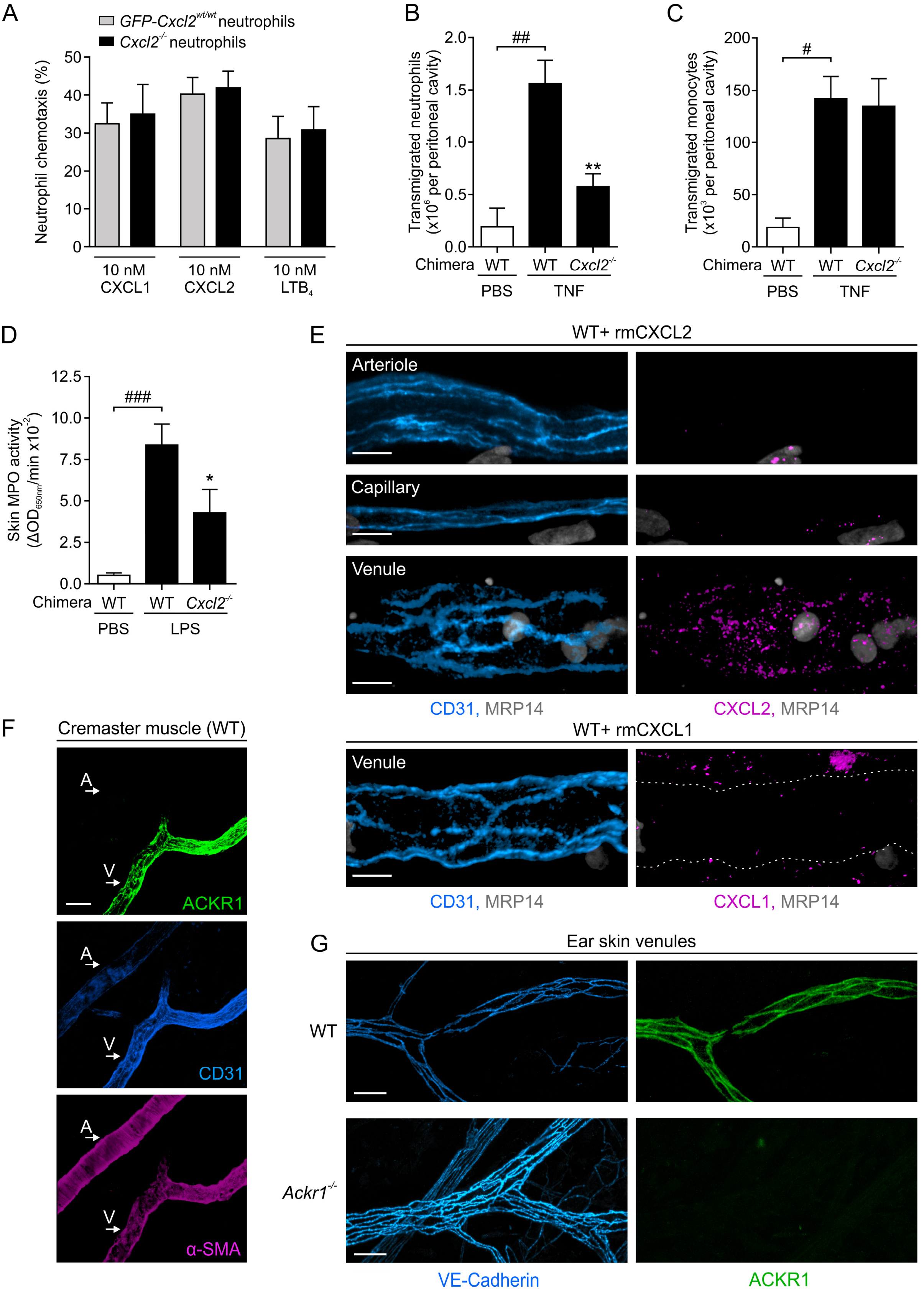

Figure S5 (related to Figure 6): The functional role of leukocyte CXCL2 in leukocyte extravasation and the expression profile of exogenous CXCL2 and CXCL1 and EC ACKR1. (A) Chemotaxis of *GFP-Cxcl2*$^{wt/wt}$ and *Cxcl2*$^{-/-}$ bone marrow neutrophils in response to CXCL1, CXCL2 or leukotriene B_4 (LTB_4) (10 nM each, 1 h) as determined by Transwell

assays (n = 3) from 3 independent experiments. (B-C) Neutrophil (B) and monocyte (C) extravasation into peritoneal cavities of WT or *Cxcl2*$^{-/-}$ chimeras injected i.p. with PBS or TNF injection (300 ng, 4 h, n = 4-10 mice per group, 4 independent experiments). (D) Neutrophil infiltration into dorsal skin sites of WT or *Cxcl2*$^{-/-}$ chimeric mice injected with PBS or lipopolysaccharide (LPS) (300 ng injected via the intradermal route, 4 h, n = 4-9 mice per group, 4 independent experiments), as quantified by myeloperoxidase (MPO) activity. (E) RmCXCL2 or rmCXCL1 was injected i.s. into WT mice and cremaster muscles were immunostained for CXCL2 or CXCL1, CD31 and MRP14. Confocal images of an arteriole, a capillary and venules are shown (representative of 8 independent experiments). Scale bars, 10 µm. Of note, levels of endogenously generated and EC-bound CXCL2 were below the detection limit of the present confocal microscopy platform. (F) Representative confocal images depicting a venule (V) and an arteriole (A) in a TNF-stimulated cremaster muscle IF stained for ACKR1, CD31 and α-SMA (representative of 3 independent experiments). Scale bar, 40 µm. (G) ACKR1 and VE-cadherin IF staining in venules of mouse ear skin in WT or *Ackr1*$^{-/-}$ mice (images are representative for 2 mice per group and 2 independent experiments). Scale bars, 30 µm. Means ± SEM, *p<0.05, **p<0.01 relative to WT chimeras and #p<0.05, ##p<0.01, ###<0.001 as indicated.

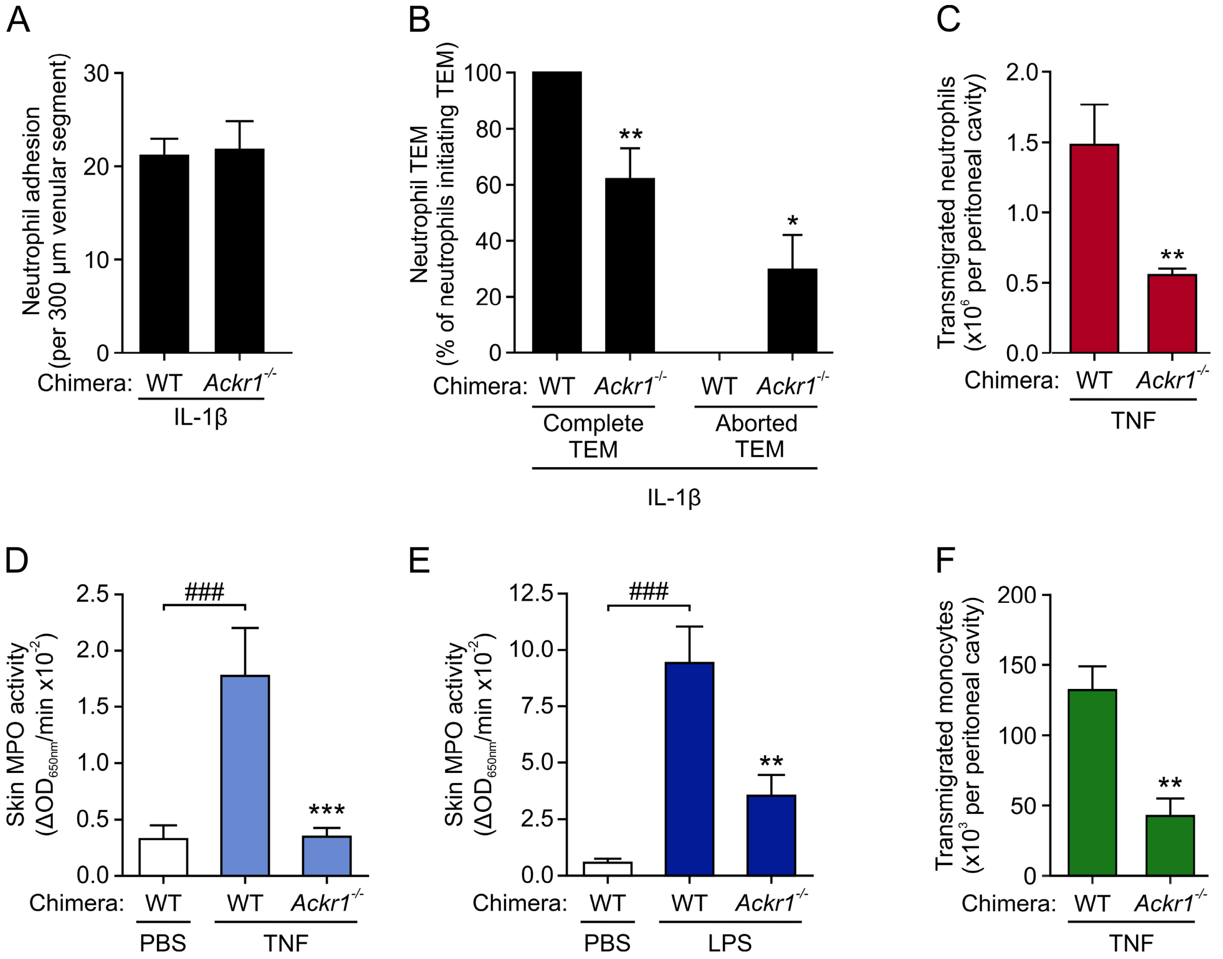

Figure S6 (related to Figure 7): EC ACKR1 facilitates neutrophil extravasation in multiple inflammatory models. (A-B) Neutrophil adhesion to ECs (A), complete TEM and aborted TEM (B) in cremaster muscle venules of WT or *Ackr1*$^{-/-}$ chimeric mice stimulated with IL-1β (50 ng, 4 h), as quantified by confocal IVM (n = 5-6 mice per group) from 11 independent experiments. (C) Neutrophil extravasation into the peritoneal cavity of WT and *Ackr1*$^{-/-}$ chimeric mice injected i.p. with TNF (300 ng, 4 h, n = 4-7 mice per group, 3 independent experiments). (D-E) Neutrophil infiltration into dorsal skin sites of WT or *Ackr1*$^{-/-}$ chimeric mice injected with PBS, TNF (D) or LPS (E) (both at 300 ng injected via the intradermal route, 4 h, n = 5-8 mice per group), as quantified by MPO activity from 4 independent experiments. (F) Monocyte extravasation in the peritoneal cavity of WT and *Ackr1*$^{-/-}$ chimeric mice injected i.p. with TNF (300 ng, 4 h, n = 4-7 mice per group, 3 independent experiments). Means ± SEM. *$p<0.05$, **$p<0.01$, ***$p<0.001$ as compared to WT chimeras and ### $p< 0.001$ as indicated.
