# Supplementary material for: Distinct Compartmentalization of the Chemokines CXCL1 and CXCL2 and the Atypical Receptor ACKR1 Determine Discrete Stages of Neutrophil Diapedesis
Source: Immunity. 2018 Dec 18;49(6):1062–1076.e6. doi: 10.1016/j.immuni.2018.09.018 (PMC6303217; doi:10.1016/j.immuni.2018.09.018)
Supplement: Document S2. Article plus Supplemental Information [file mmc6.pdf]

Immunity

Article

Distinct Compartmentalization of the Chemokines CXCL1 and CXCL2 and the Atypical Receptor ACKR1 Determine Discrete Stages of Neutrophil Diapedesis

Graphical Abstract

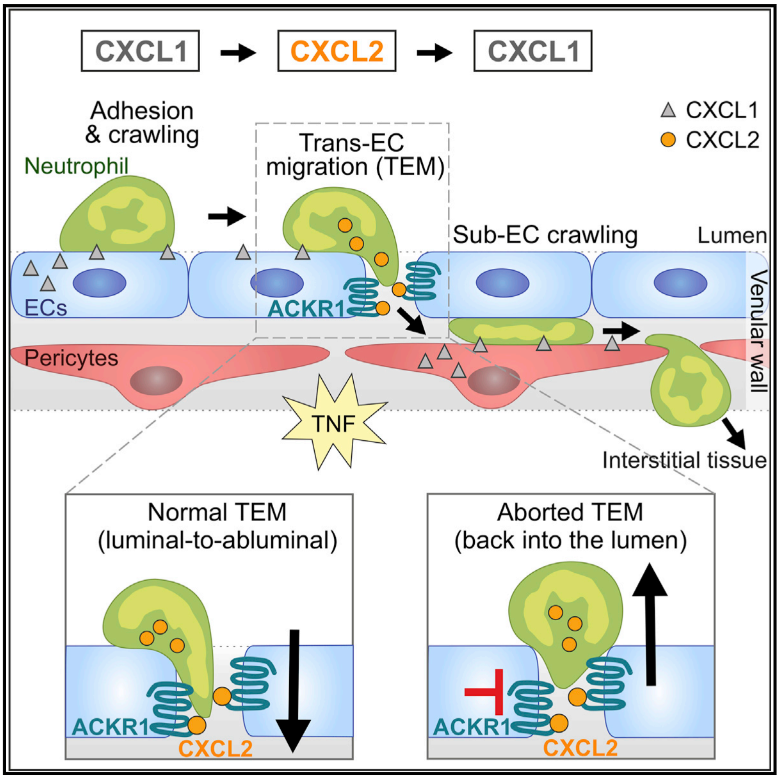

Highlights

- The CXCR2 ligands CXCL1 and CXCL2 play distinct roles in neutrophil diapedesis
- Endothelial cell (EC)- and pericyte-derived CXCL1 mediate neutrophil crawling
- CXCL2 is neutrophil derived and is retained at EC junctions by ACKR1
- ACKR1-CXCL2 axis is critical for unidirectional paracellular neutrophil TEM *in vivo*

Authors

Tamara Girbl, Tchern Lenn, Lorena Perez, ..., Mathieu-Benoit Voisin, Antal Rot, Sussan Nourshargh

Correspondence

s.nourshargh@qmul.ac.uk

In Brief

Girbl et al. find that sequential interactions with the chemokines CXCL1 and CXCL2 guide neutrophil crawling and subsequent migration through venular walls during inflammation. Transmigrating neutrophils promoted unidirectional luminal-to-abluminal migration by depositing CXCL2 on the chemokine receptor ACKR1 at endothelial junctions, providing a paradigm of self-guided migration.

Girbl et al., 2018, Immunity *49*, 1062–1076
December 18, 2018 © 2018 The Authors. Published by Elsevier Inc.
https://doi.org/10.1016/j.immuni.2018.09.018

CellPress

Immunity
Article

Distinct Compartmentalization of the Chemokines CXCL1 and CXCL2 and the Atypical Receptor ACKR1 Determine Discrete Stages of Neutrophil Diapedesis

Tamara Girbl,[1] Tchern Lenn,[1] Lorena Perez,[1] Loïc Rolas,[1] Anna Barkaway,[1] Aude Thiriot,[3] Carlos del Fresno,[4] Eleanor Lynam,[1] Elin Hub,[1] Marcus Thelen,[5] Gerard Graham,[6] Ronen Alon,[7] David Sancho,[4] Ulrich H. von Andrian,[3] Mathieu-Benoit Voisin,[1] Antal Rot,[1,2,8] and Sussan Nourshargh[1,2,9,*]
[1]Centre for Microvascular Research, William Harvey Research Institute, Barts and The London School of Medicine and Dentistry, Queen Mary University of London, London EC1M 6BQ, UK
[2]Centre for Inflammation and Therapeutic Innovation, Barts and The London School of Medicine and Dentistry, Queen Mary University of London, London EC1M 6BQ, UK
[3]Department of Microbiology and Immunobiology and HMS Center for Immune Imaging, Harvard Medical School, Boston, MA 02115, USA
[4]Immunobiology Laboratory, Centro Nacional de Investigaciones Cardiovasculares (CNIC), Madrid 28029, Spain
[5]Institute for Research in Biomedicine, Università della Svizzera Italiana, Bellinzona 6500, Switzerland
[6]Institute of Infection, Immunity and Inflammation, University of Glasgow, Glasgow G12 8TA, UK
[7]Department of Immunology, The Weizmann Institute of Science, Rehovot 7610001, Israel
[8]Institute for Cardiovascular Prevention, Ludwig-Maximilians University, Munich 80336, Germany
[9]Lead Contact
*Correspondence: s.nourshargh@qmul.ac.uk
https://doi.org/10.1016/j.immuni.2018.09.018

SUMMARY

Neutrophils require directional cues to navigate through the complex structure of venular walls and into inflamed tissues. Here we applied confocal intravital microscopy to analyze neutrophil emigration in cytokine-stimulated mouse cremaster muscles. We identified differential and non-redundant roles for the chemokines CXCL1 and CXCL2, governed by their distinct cellular sources. CXCL1 was produced mainly by TNF-stimulated endothelial cells (ECs) and pericytes and supported luminal and sub-EC neutrophil crawling. Conversely, neutrophils were the main producers of CXCL2, and this chemokine was critical for correct breaching of endothelial junctions. This pro-migratory activity of CXCL2 depended on the atypical chemokine receptor 1 (ACKR1), which is enriched within endothelial junctions. Transmigrating neutrophils promoted a self-guided migration response through EC junctions, creating a junctional chemokine "depot" in the form of ACKR1-presented CXCL2 that enabled efficient unidirectional luminal-to-abluminal migration. Thus, CXCL1 and CXCL2 act in a sequential manner to guide neutrophils through venular walls as governed by their distinct cellular sources.

INTRODUCTION

Neutrophils form the principal cellular arm of innate immunity and are, as such, the host's first line of protection in response to infections and injury. Central to the neutrophils' functions is their ability to rapidly exit the vascular compartment and migrate within the extravascular tissue toward the core of an inflammatory insult. This exquisitely coordinated behavior can be broadly split into three stages: (1) neutrophil migratory responses on the luminal aspect of venules, (2) migration through venular walls, and finally, (3) directional interstitial motility (Nourshargh et al., 2010). In line with the universally accepted concept of directed cell migration, these phases of neutrophil movement are considered to be driven by locally presented chemotactic cues. Indeed, there is ample evidence for the ability of chemokines to trigger rapid integrin-mediated attachment to and crawling of neutrophils on venular endothelial cells (ECs) (Nourshargh and Alon, 2014). Furthermore, analysis of leukocyte motility within inflamed tissues by confocal intravital microscopy (IVM) has revealed the existence of multistep attraction cascades involving an amplifying feed-forward mechanism as choreographed by different types of chemoattractants (Kienle and Lämmermann, 2016). In contrast to our understanding of luminal and tissue migratory responses, less is known about the nature and localization of directional cues that guide neutrophils through the complex bicellular structures of venular walls composed of ECs and pericytes, a phenomenon that has been investigated here.

The exit of neutrophils from the vascular compartment requires breaching of the venular endothelium, followed by crossing the pericyte sheath that is embedded within the venular basement membrane (Nourshargh et al., 2010). Transendothelial cell migration (TEM) commonly occurs in a paracellular manner as mediated by junctionally expressed molecules such as CD31, members of the junctional adhesion molecule (JAM) family, VE-Cadherin, and CD99 (Nourshargh and Alon, 2014). Furthermore, once in the sub-EC space, neutrophils exhibit significant crawling on pericyte processes, a response that appears to represent the neutrophil's quest for permissive exit portals (Proebstl et al., 2012). Indeed, full breaching of venular walls predominantly occurs via gaps between adjacent

 © 2018 The Authors. Published by Elsevier Inc.
This is an open access article under the CC BY license (http://creativecommons.org/licenses/by/4.0/).

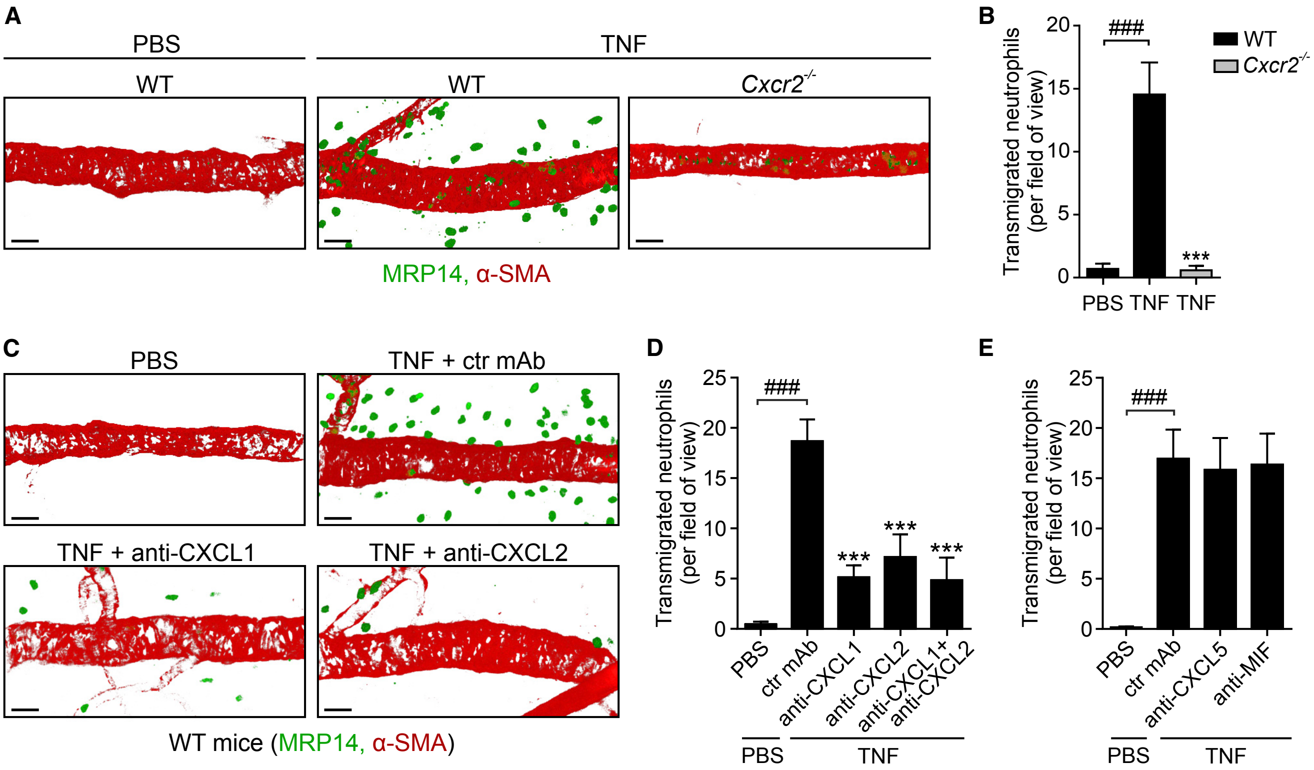

Figure 1. TNF-Induced Neutrophil Migration Is Dependent on Both CXCL1 and CXCL2

WT mice pre-treated intrascrotally (i.s.) with control (ctr) or blocking mAbs or *Cxcr2*$^{-/-}$ mice were subjected to i.s. injections of PBS or TNF. The cremaster muscles were immunostained for MRP14 (neutrophils) and α-SMA (pericytes) and analyzed for neutrophil infiltration by confocal microscopy. (A) and (C) are representative images and (B), (D), and (E) show quantifications (n = 4–10 mice per group) from 5–6 independent experiments. Means ± SEM, ***p < 0.001 as compared to TNF-treated ctrls and ###p < 0.001 as indicated. Scale bars, 30 μm. See also Figure S1.

pericytes and regions within the venular basement membrane that express reduced levels of certain matrix proteins (Proebstl et al., 2012; Wang et al., 2006). Despite our growing understanding of the intricacies of neutrophil behavior within venular walls, and certain aspects of the associated molecular machinery, at present little is known about the patterning of directional cues that sequentially guide neutrophils through the endothelium and the pericyte layer. Here we set to define the molecular determinants of neutrophil migration through ECs and pericytes in inflamed cremaster muscles *in vivo* using high-resolution confocal IVM. We found an absolute requirement for CXCR2 in TNF-induced neutrophil emigration and identified the two CXCR2 ligands CXCL1 and CXCL2 as the principal directional cues that mediate this response. Despite their highly homologous structures (~90% in amino acid sequence), CXCL1 and CXCL2 acted specifically and in a non-redundant and sequential manner to guide neutrophils through venular walls as governed by their distinct cellular sources. Of note, CXCL2 was almost exclusively derived from neutrophils, presenting a paradigm whereby transmigrating neutrophils promote a CXCL2-dependent self-guided migration response through EC junctions. This mechanism entrails the ability of endothelial atypical chemokine receptor 1 (ACKR1) enriched within junctions to retain extrinsic CXCL2, thus creating a junctional chemokine "depot" required for efficient unidirectional luminal-to-abluminal neutrophil migration.

RESULTS

TNF-Induced Neutrophil Migration Is Dependent on Both CXCL1 and CXCL2

To investigate how directional cues guide neutrophils through venular walls, we analyzed a robust acute inflammatory reaction elicited by locally administered TNF within the mouse cremaster muscle. Initial work aimed to identify the endogenous neutrophil chemotactic cues generated in this model. We found that while locally injected TNF (300 ng, 4 hr) induced a strong neutrophil infiltration response in wild-type (WT) mice, this was totally inhibited in CXCR2-deficient animals (Figures 1A and 1B). Murine CXCR2 is the receptor for several ELR^{+} chemokines, namely two potent neutrophil chemoattractants (CXCL1 and CXCL2) as well as CXCL3, CXCL5, and CXCL7 and a non-chemokine macrophage migration inhibitory factor (MIF) (Zlotnik and Yoshie, 2012). Co-injection of TNF with blocking mAbs against CXCL1 or CXCL2 led to >60% inhibition of neutrophil accumulation, as compared to tissues injected with an isotype-matched control mAb (Figures 1C and 1D). Co-injection of TNF simultaneously with both anti-CXCL1 and anti-CXCL2 mAbs did not cause a greater inhibition than that noted with either mAb alone (Figure 1D). Similar outcomes were observed in a TNF-induced peritonitis model (Figure S1). Antibody blockade of two other CXCR2 ligands, CXCL5 and MIF, did not impact TNF-induced neutrophil infiltration into cremaster muscles (Figure 1E).

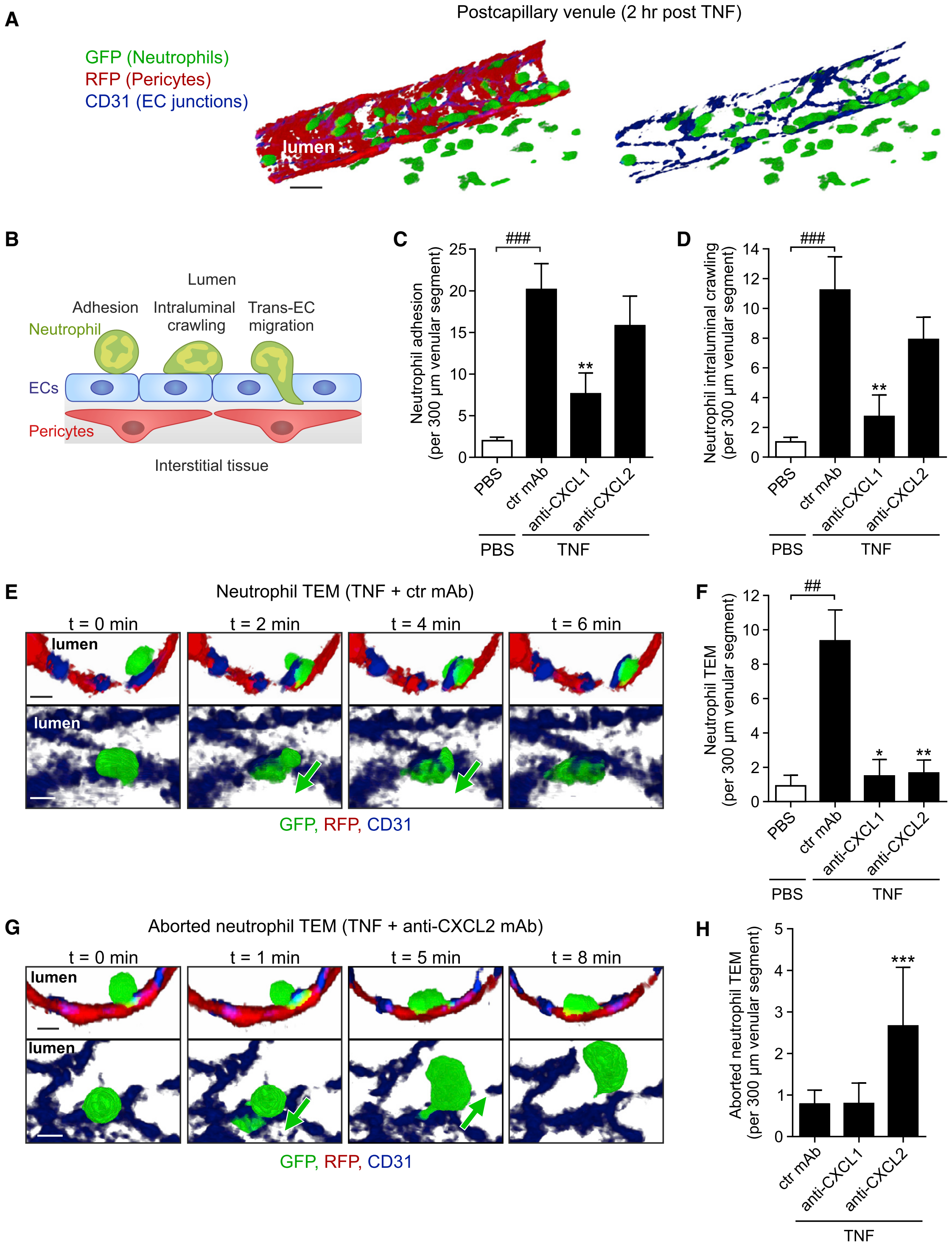

Figure 2. TNF-Elicited CXCL1 and CXCL2 Support Distinct Phases of Neutrophil-EC Interactions

Lyz2-EGFP-ki;Acta2-RFPcherry-Tg mice, subjected to *in vivo* CD31 labeling, were treated with ctr or blocking mAbs (i.v. 10 min prior to TNF in C and D and i.s. 100 min post TNF in E–H) and neutrophil responses in cremaster muscles injected locally with PBS or TNF quantified by confocal IVM.

(A) Illustrative images of the employed IVM model (scale bar, 20 μm).

(B) Scheme depicting neutrophil responses quantified in (C)–(H).

(C and D) Quantification of neutrophil adhesion and intraluminal crawling (n = 5–6 mice per group, 23 independent experiments).

(E) Time-lapse IVM images (Video S1) of a neutrophil TEM response in a TNF-stimulated tissue showing a neutrophil migrating from the lumen (0 min) through an EC junction (2–4 min) into the sub-EC space (6 min). Representative of 11 independent experiments; cross sections, top; luminal views, bottom; scale bars, 5 μm.

(legend continued on next page)

Collectively, these findings show that TNF-induced neutrophil migration *in vivo* is dependent on the generation of CXCL1 and CXCL2 in the tissue and involves signaling via their cognate receptor CXCR2. Because mAb blockade targeting either or both chemokines simultaneously achieved the same level of inhibition, we hypothesized that CXCL1 and CXCL2 may guide neutrophils through venular walls by acting sequentially along the cell migratory pathway.

TNF-Elicited CXCL1 and CXCL2 Support Distinct Phases of Neutrophil-EC Interactions

To explore the roles of TNF-induced endogenous CXCL1 and CXCL2 in neutrophil migration through venular walls, the effects of neutralizing mAbs on key phases were investigated by confocal IVM. The latter is a model developed and optimized for analyzing neutrophil-vessel wall interactions, enabling direct and simultaneous tracking of neutrophil responses in relation to ECs and pericytes in 3D with high spatiotemporal resolution (Figure 2A; Proebstl et al., 2012). We employed the compound reporter mouse *Lyz2-EGFP-ki;Acta2-RFPcherry-Tg* that displays GFP^+ myeloid cells and RFP^+ smooth muscle cells and pericytes (Proebstl et al., 2012), applying image capture settings that selectively detect GFP^{bright} neutrophils, and labeling EC junctions *in vivo* using locally applied non-blocking Alexa Fluor 647-anti-CD31 mAb (Woodfin et al., 2011). Initially we investigated the roles of CXCL1 and CXCL2 in early luminal responses of neutrophil adhesion and crawling in post-capillary venules (Figure 2B). With respect to both parameters, local injection of TNF (300 ng; tissues imaged within the 2–4 hr *in vivo* test period) induced significant responses as compared to PBS-treated tissues (Figures 2C and 2D). Animals injected with intravenous (i.v.) anti-CXCL1 mAb (~10 min prior to administration of TNF) exhibited significantly reduced neutrophil adhesion and crawling, as compared to mice treated with a control mAb (>62% for both). In contrast, using the same protocol, CXCL2 blockade had no significant effect on neutrophil adhesion or crawling (Figures 2C, 2D, S2A, and S2B).

After phases of luminal adhesion and crawling, neutrophils breach the EC barrier by the active step of TEM (Nourshargh and Alon, 2014). In line with our previous work (Woodfin et al., 2011), neutrophil TEM was predominantly paracellular (i.e., via EC junctions; ~96%) and occurred rapidly (~6 min) in a luminal-to-abluminal direction (Figure 2E and Video S1). To assess the roles of CXCL1 and CXCL2 in neutrophil TEM, we tested locally applied blocking reagents in order to target post luminal events. For this purpose, blocking or control mAbs were injected intrascrotally (i.s.) 100 min post injection of TNF, thus allowing the development of a robust initial adhesion and diapedesis response. Within this model, anti-CXCL1 mAbs again suppressed luminal adhesion (37.7%) but also exerted a profound inhibition of neutrophil TEM, as compared to responses in control mAb-treated tissues (~84% inhibition; Figure 2F). However, under these conditions, the fraction of neutrophils that established a robust crawling response on ECs displayed a similar percentage of neutrophil TEM to that noted in TNF-stimulated tissues treated with a control mAb (~17% and ~25%, respectively; Figure S2C). These results indicate that while endogenous CXCL1 plays a critical role in supporting luminal neutrophil firm adhesion, it does not mediate neutrophil TEM per se, and as such, the inhibitory effect of the anti-CXCL1 mAb on TEM is due to suppression of preceding luminal responses. In contrast, but in agreement with the data obtained using i.v. anti-CXCL2 mAb, locally injected anti-CXCL2 mAb exerted no suppression of neutrophil adhesion or luminal crawling (data not shown) but induced almost total inhibition of neutrophil TEM (Figure 2F). Indeed, in tissues treated with the anti-CXCL2 mAb, a significant number of luminal neutrophils initiated TEM by extending protrusions through EC junctions, but then retracted and exhibited reverse motility back into the blood circulation (Figures 2G and 2H; Video S2). This disrupted mode of TEM that we have termed "aborted" TEM rarely occurred in TNF-stimulated tissues treated with control or anti-CXCL1 mAbs (~2% and ~8% of crawling neutrophils, respectively) but was notably evident in tissues subjected to CXCL2 blockade (~15% of crawling cells, $p < 0.01$, n = 5–10 mice per group; Figure 2H). These results indicate an important role for CXCL2 in providing directional cues for neutrophils within EC junctions and hence mediating persistent migration of neutrophils from the apical to basolateral aspect of ECs *in vivo*.

Together, these data identify distinct roles for endogenously generated CXCL1 and CXCL2 in neutrophil migration through TNF-inflamed venular walls *in vivo*, supporting luminal neutrophil adhesion and crawling, and luminal-to-abluminal TEM, respectively.

Neutrophil-Pericyte Interactions Are Selectively Mediated by Endogenous CXCL1

Post TEM, neutrophils exhibit substantial sub-EC crawling on pericyte processes before fully exiting the venular wall via gaps between adjacent pericytes (Figures 3A and 3B; Video S3; Proebstl et al., 2012). To investigate the potential involvement of CXCL1 and CXCL2 in this response, we injected blocking reagents locally, as employed for the analysis of neutrophil TEM. As the anti-chemokine mAbs were injected 100 min post injection of TNF, the protocol enabled undisturbed passage of a large number of neutrophils through the endothelium and into the sub-EC space (average of ~15 neutrophils per venular segment in all conditions studied), allowing rigorous analysis of neutrophil-pericyte interactions. Tracking of cells in the sub-EC space showed that in contrast to control tissues, the majority of neutrophils in anti-CXCL1 mAb-treated mice exhibited no crawling and indeed appeared stationary during the observation period of 2 hr (Figures 3C and 3D). CXCL2 blockade exerted no such effect (Figure 3D). Furthermore, anti-CXCL1 mAb significantly reduced

(F) Quantifications of neutrophil TEM (n = 4–11 mice per group, 27 independent experiments).

(G) IVM images (Video S2) of an aborted TEM response in a mouse treated with local TNF+anti-CXCL2 mAb. The images show a luminal neutrophil extending a protrusion through an EC junction (1 min), retracting the protrusion, and re-entering the circulation (5–8 min). Representative of 6 independent experiments; cross sections and luminal views; scale bars, 5 μm.

(H) Quantification of aborted neutrophil TEM (n = 4–11 mice per group, 21 independent experiments). Means ± SEM, *$p < 0.05$, **$p < 0.01$, ***$p < 0.001$ as compared to TNF-treated ctrls and ##$p < 0.01$, ###$p < 0.001$ as indicated. See also Figure S2.

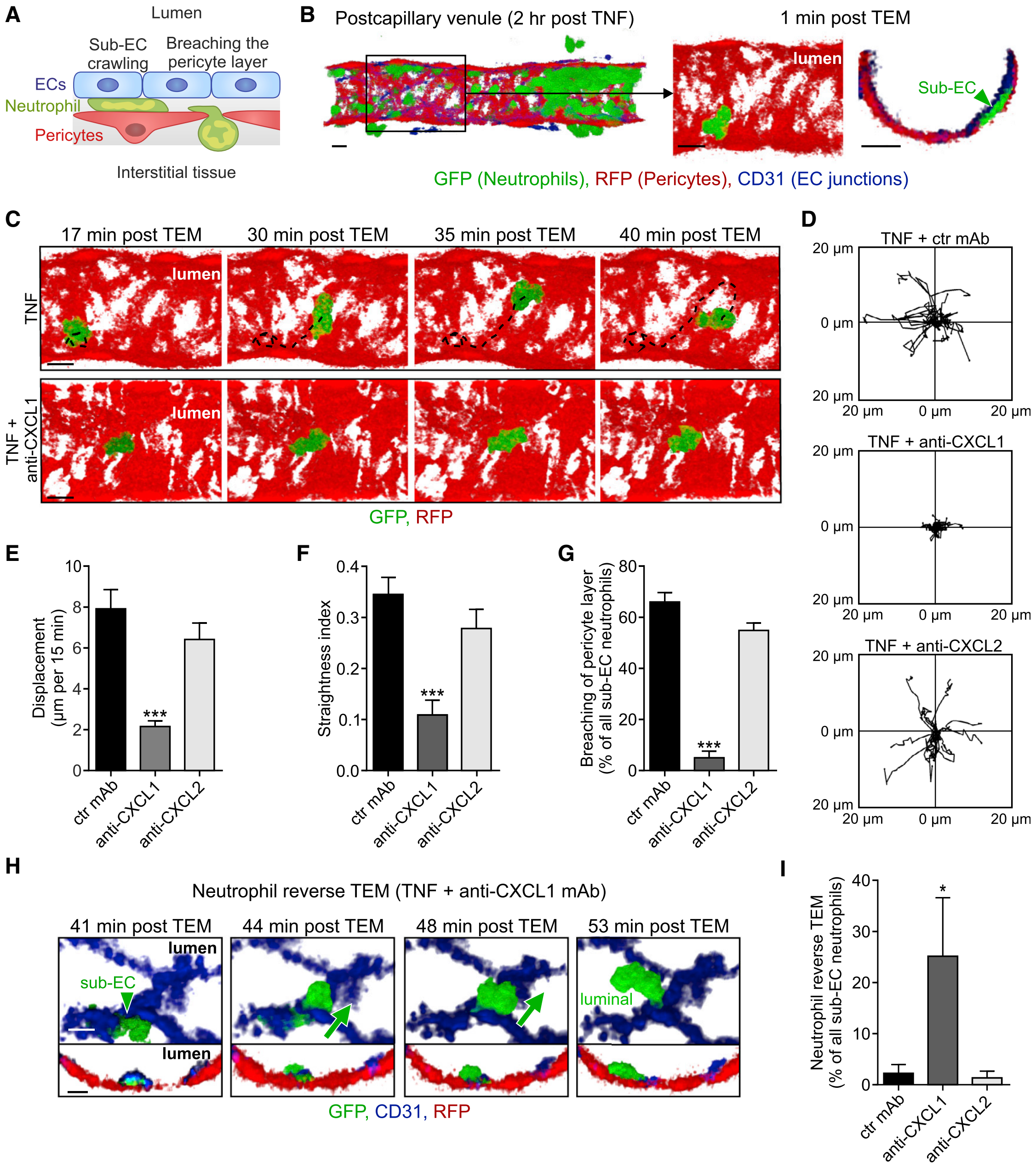

Figure 3. Neutrophil-Pericyte Interactions Are Selectively Mediated by Endogenous CXCL1

Lyz2-EGFP-ki;Acta2-RFPcherry-Tg mice, subjected to *in vivo* CD31 labeling, were stimulated locally with TNF and 100 min later i.s. injected with ctr or blocking mAbs, as indicated.

(A) Neutrophil responses quantified in cremasteric venules by confocal IVM in (C)–(G).

(B) Representative confocal IVM luminal and cross-sectional views depicting a neutrophil localized between TNF-stimulated ECs and pericytes 1 min post TEM.

(C) Time-lapse IVM images (Video S3) showing a neutrophil crawling on pericytes (tracks, dashed lines) in a TNF-stimulated tissue (top) and the inhibition of this response in tissues treated with anti-CXCL1 mAb (bottom).

Scale bars in (B) and (C), 10 μm.

(D–G) Crawling profiles of neutrophils on pericytes (20 cells per group for clarity) (D) as normalized for their origin and associated quantifications of displacement (E), straightness index (displacement/track length) (F), and breaching of the pericyte layer (G).

(H) Time-lapse confocal IVM images (Video S4) illustrating neutrophil reverse TEM in a tissue treated with TNF+anti-CXCL1 mAb (luminal and cross-sectional views; scale bars, 5 μm).

(I) Quantifications of neutrophil reverse TEM. Images are representative of 5–10 independent experiments and quantifications (n = 5–10 mice per group) involve 20 independent experiments.

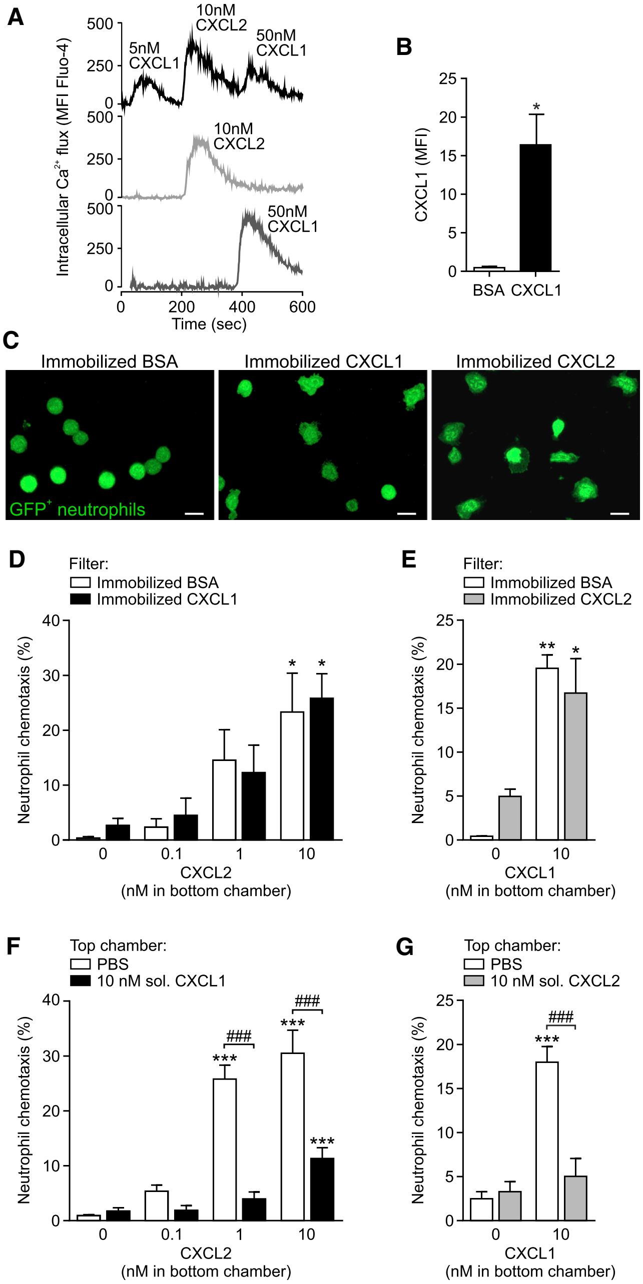

Figure 4. Neutrophils Can Respond to Sequential Exposure to CXCL1 and CXCL2

(A) Intracellular Ca^{2+} flux in neutrophils as induced by serial or single stimulations with chemokines representative of 4 independent experiments.

(B) CXCL1 IF staining on Transwell filters coated with BSA or CXCL1 (n = 3) from 3 independent experiments.

(C) Confocal images of GFP^{+} neutrophils seeded on BSA-, CXCL1-, or CXCL2-coated Transwell filters representative of 3 independent experiments. Scale bars, 10 μm.

(D–G) Chemotaxis of neutrophils exposed to immobilized CXCL1 or CXCL2 (D and E) or soluble CXCL1 or CXCL2 (F and G) into bottom chambers containing indicated chemokines (1 hr at 37°C; n = 4) from 2–4 independent experiments. Means ± SEM, *$p < 0.05$, **$p < 0.01$, ***$p < 0.001$ as compared to corresponding responses without chemokines in bottom chambers and ###$p < 0.001$ as indicated.

See also Figure S3.

the displacement (Figure 3E) and straightness index (displacement/track length; Figure 3F) of sub-EC crawling neutrophils, as compared to control mAb treatment, while CXCL2 blockade failed to exert any such effects (Figures 3E and 3F).

The inhibitory impact of anti-CXCL1 mAb on neutrophil motility on pericytes resulted in a substantial retention of neutrophils within venular walls with only a small fraction of neutrophils breaching the pericyte layer as compared to responses quantified in control tissues (Figure 3G). However, the number of retained neutrophils dropped gradually over the 2 hr observation period as a significant fraction of neutrophils in the sub-EC space reverse migrated through EC junctions and re-entered the blood circulation (>25%; Figures 3H and 3I; Video S4). No such effect was seen in anti-CXCL2 mAb-treated tissues.

Overall, these results demonstrate that TNF-induced CXCL1, but not CXCL2, is an essential mediator of neutrophil motility on pericytes and promotes neutrophil passage through the pericyte layer, a critical step in neutrophil recruitment to inflamed tissues (Proebstl et al., 2012).

Neutrophils Can Respond to Sequential Exposures to CXCL1 and CXCL2

Having identified that neutrophil migration through venular walls is mediated by sequential actions of CXCL1, CXCL2, and again CXCL1, we sought to directly investigate the ability of neutrophils in responding to serial activation of CXCR2 by these agonists. Specifically, we wished to identify the conditions under which neutrophils exposed to CXCL1 or CXCL2 could still sense gradients of the other chemokine. CXCR2 is a Gαi-coupled receptor and its ligation by corresponding agonists can trigger multiple downstream signaling pathways including the activation of PLCβ and PI3Kγ (Stadtmann and Zarbock, 2012). The former elicits enhanced intracellular Ca^{2+} and diacylglycerol production that are critical for rapid integrin activation and neutrophil arrest, while activation of PI3Kγ promotes AKT phosphorylation, a signaling pathway required for chemotaxis (Stadtmann and Zarbock, 2012). Our initial studies showed that CXCL1 and CXCL2 are equipotent in stimulating mouse neutrophils to exhibit Ca^{2+} flux and adhesion to ICAM-1-coated plates (Figures S3A and S3B). Both chemokines also stimulated neutrophil AKT phosphorylation and chemotaxis (Figures S3C and S3D). These results are consistent with the fact that when administered exogenously, CXCL1 and CXCL2 can induce similar neutrophil transmigration responses (Zhang et al., 2001).

We next explored the ability of neutrophils to respond to sequential stimulations by the two agonists under distinct conditions of CXCL1 and CXCL2 encounter. Initially, aiming to mimic the cascade of responses noted *in vivo*, Fluo-4-loaded bone marrow neutrophils were treated in a consecutive manner with CXCL1, CXCL2, and finally with CXCL1 again. The subsequent exposure to the same concentration of agonist resulted in notably reduced Ca^{2+} signals as compared to responses detected in cell samples stimulated with one agonist only (data not shown). In contrast, with increasing concentrations of agonists, neutrophils were able to exhibit robust, rapid, and transient increases in intracellular Ca^{2+} following serial activations (Figure 4A). However, as found before, cells stimulated with soluble CXCL1 and/or CXCL2 showed reduced intracellular Ca^{2+} flux to subsequent agonist stimulations, as compared to responses

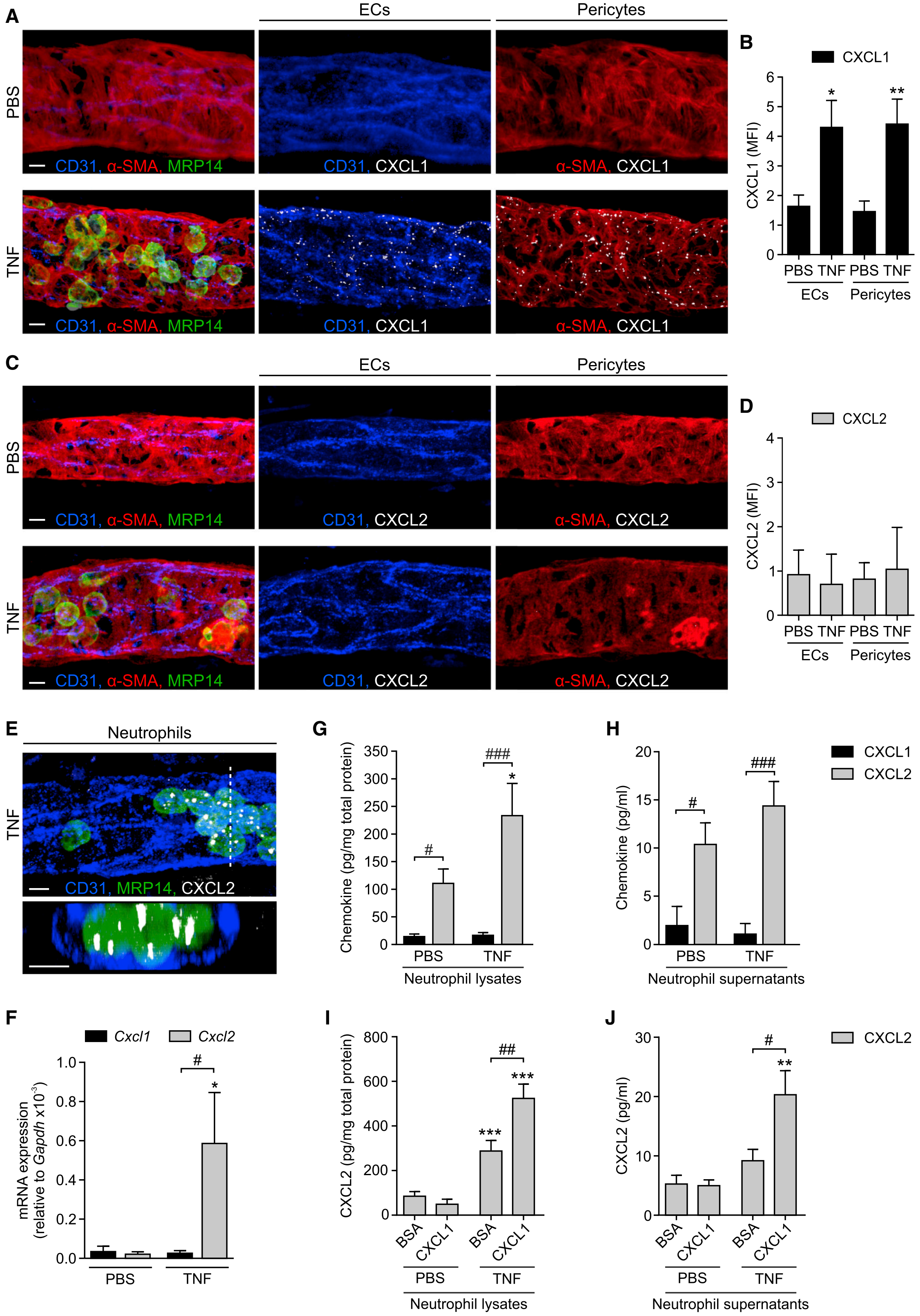

Figure 5. CXCL1 and CXCL2 Are Differentially Expressed in TNF-Stimulated Tissues

(A–E) WT mice were treated i.s. with PBS or TNF and cremaster muscles were IF stained for CXCL1 or CXCL2 and CD31 (ECs), α-SMA (pericytes), and MRP14 (neutrophils). Representative confocal images of venules showing CXCL1 (A) or CXCL2 (C) staining within EC and pericyte isosurface masks and quantifications

(legend continued on next page)

elicited following a single chemokine exposure (Figure S3E). To further mimic the *in vivo* scenario, and in line with the accepted paradigm that luminal neutrophil adhesion and crawling is mediated by chemokines expressed on the cell surface of ECs (Nourshargh and Alon, 2014), we tested the impact of immobilized CXCL1 on CXCL2-induced neutrophil chemotaxis. GFP^+ neutrophils (isolated from *Lyz2-EGFP-ki* mice) were seeded onto Transwell filters coated with either BSA or CXCL1, and the bottom chambers were filled with medium containing CXCL2 (0.1–10 nM). The presentation of biologically active CXCL1 on chemokine-coated filters was confirmed by immunofluorescence (IF) staining and the flattened morphology of neutrophils, as compared to BSA-coated filters (Figures 4B and 4C). Strikingly, neutrophils migrating through BSA- and CXCL1-coated filters exhibited identical chemotactic responses to CXCL2 (Figure 4D). Similarly, exposure of neutrophils to immobilized CXCL2 (presentation and activity again confirmed by IF staining and neutrophil morphology; Figures S3F and 4C) did not affect neutrophil chemotaxis into CXCL1-containing bottom chambers (Figure 4E). In contrast, and in line with our Ca^{2+} flux data, neutrophils exposed to soluble CXCL1 or CXCL2 (both 10 nM) in the upper chamber of Transwell plates showed profoundly suppressed migration to either CXCL2 or CXCL1, respectively, in the lower chamber (Figures 4F and 4G).

Collectively, the data illustrate that neutrophils are able to respond to sequential stimulations by CXCL1 and CXCL2, especially when the first chemokine is in an immobilized state. Thus, the distinct and sequential roles of these chemokines in guiding neutrophils through venular walls *in vivo* may be governed by their potentially differential temporal and spatial presentation to migrating neutrophils.

CXCL1 and CXCL2 Are Differentially Expressed in TNF-Stimulated Tissues

To investigate the cellular source and distribution of CXCL1 and CXCL2 generated within TNF-stimulated venules, in initial studies, permeabilized whole-mounted tissues were analyzed by IF staining. While control PBS-treated tissues showed almost no CXCL1 signal in CD31-labeled ECs and α-SMA-stained pericytes (also identified by their morphology and anatomical location), CXCL1 expression was notably enhanced in both cell types in TNF-stimulated samples (Figures 5A and 5B). This staining was in a punctate pattern, an expression profile that may represent intracellular CXCL1 vesicular depots *en route* to secretion and/or cell surface presentation, and in ECs appeared evenly distributed in junctional and non-junctional regions (mean fluorescence intensity [MFI] of CXCL1 IF staining: 4.9 and 4.6, respectively, n = 3; Figure S4A). The vesicular profile of CXCL1 is in agreement with previous works reporting intracellular chemokine puncta in the context of CCL2 in TNF-stimulated ECs (Shulman et al., 2011) and CCL21 in lymphatic ECs (Vaahtomeri et al., 2017). In contrast to CXCL1, CXCL2 was hardly detectable in venular ECs and pericytes in both control and TNF-stimulated tissues (Figures 5C and 5D). Furthermore, in TNF-stimulated tissues, vascular wall cells were an abundant source of *Cxcl1* (but not *Cxcl2*) mRNA expression *in vivo*, as detected and quantified by RNA fluorescence *in situ* hybridization (FISH; Figures S4B–S4E). Since *Cxcl1* gene expression in stimulated ECs has previously been reported (Chou et al., 2010; Li et al., 2016), we sought further evidence for the striking differences between the cellular localization of CXCL1 and CXCL2 in the context of pericytes. For this purpose, we established a method of isolating and culturing venular cremaster muscle pericytes from *Acta2-RFPcherry-Tg* mice (Figures S4F and S4G). In line with our *in vivo* results, TNF stimulation (4 hr) of cultured pericytes led to a robust release of CXCL1 but low levels of CXCL2, as analyzed by ELISA (Figure S4H).

While collectively these results provided conclusive evidence for CXCL1, but not CXCL2, being predominantly derived from ECs and pericytes, the cellular source of CXCL2 within venular walls remained elusive. Here, as IF stainings indicated abundant expression of CXCL2 in luminal neutrophils in TNF-stimulated tissues (Figure 5E), we extended this observation to mRNA (real-time PCR) and protein (ELISA) analysis of CXCL2, as compared to CXCL1, using isolated murine neutrophils. While blood neutrophils obtained from control PBS-stimulated mice showed low *Cxcl1* and *Cxcl2* mRNA levels, neutrophils isolated from mice subjected to local i.s. TNF (2–3 hr) exhibited strong induction of *Cxcl2*, but not *Cxcl1*, mRNA (Figure 5F). In contrast, *Cxcl1* (but not *Cxcl2*) transcripts were strongly increased in cultured cytokine-stimulated lung ECs and primary cremaster muscle pericytes (~12- and ~47-fold increase, as compared to unstimulated cells, respectively). In line with the mRNA results, analysis of protein levels by ELISA in supernatants and cell lysates derived from unstimulated and TNF-stimulated (1 nM, 1 hr) neutrophils revealed low levels of CXCL1 (Figures 5G and 5H). In contrast, mouse neutrophils expressed CXCL2 under basal conditions (potentially in pre-formed stores) that was further increased in TNF-stimulated cells (Figures 5G and 5H). Furthermore, TNF-stimulated neutrophils seeded on immobilized CXCL1 showed an even greater increase in levels of cell-associated and released CXCL2, as compared to TNF-treated cells placed onto immobilized BSA (Figure 5I and 5J). These findings support the notion that CXCL1 derived from TNF-stimulated ECs and presented to neutrophils on the luminal aspect of the endothelium can promote CXCL2 generation and release by adherent and/or crawling neutrophils.

Collectively, these data show differential sources of CXCL1 and CXCL2 in inflamed venules, with CXCL1 being primarily

of CXCL1 (B) and CXCL2 (D) in ECs or pericytes, in terms of MFIs (n = 4 mice per group) from 4 independent experiments. (E) Confocal abluminal and cross-sectional images (acquired along the dashed line and presented at 90° rotation) of a venule showing overall CXCL2 staining representative of 4 independent experiments.

(F) *Cxcl1* and *Cxcl2* mRNA levels relative to *Gapdh* in circulating neutrophils 2–3 hr after i.s. PBS or TNF injection as determined by real-time PCR (n = 4–5 mice per group, 2 independent experiments).

(G–J) Purified mouse bone marrow neutrophils were treated with PBS or TNF (1 nM, 1 hr) on uncoated (G, H), BSA-coated, or CXCL1-coated wells (I, J). CXCL1 and/or CXCL2 levels in lysates (G and I) and supernatants (H and J) as quantified by ELISA (n = 4–12) from 2–4 independent experiments. Means ± SEM, *$p < 0.05$, **$p < 0.01$, ***$p < 0.001$ as compared to corresponding PBS-treated ctrls and #$p < 0.05$, ##$p < 0.01$, ###$p < 0.001$, as indicated. Scale bars, 5 μm.

See also Figure S4.

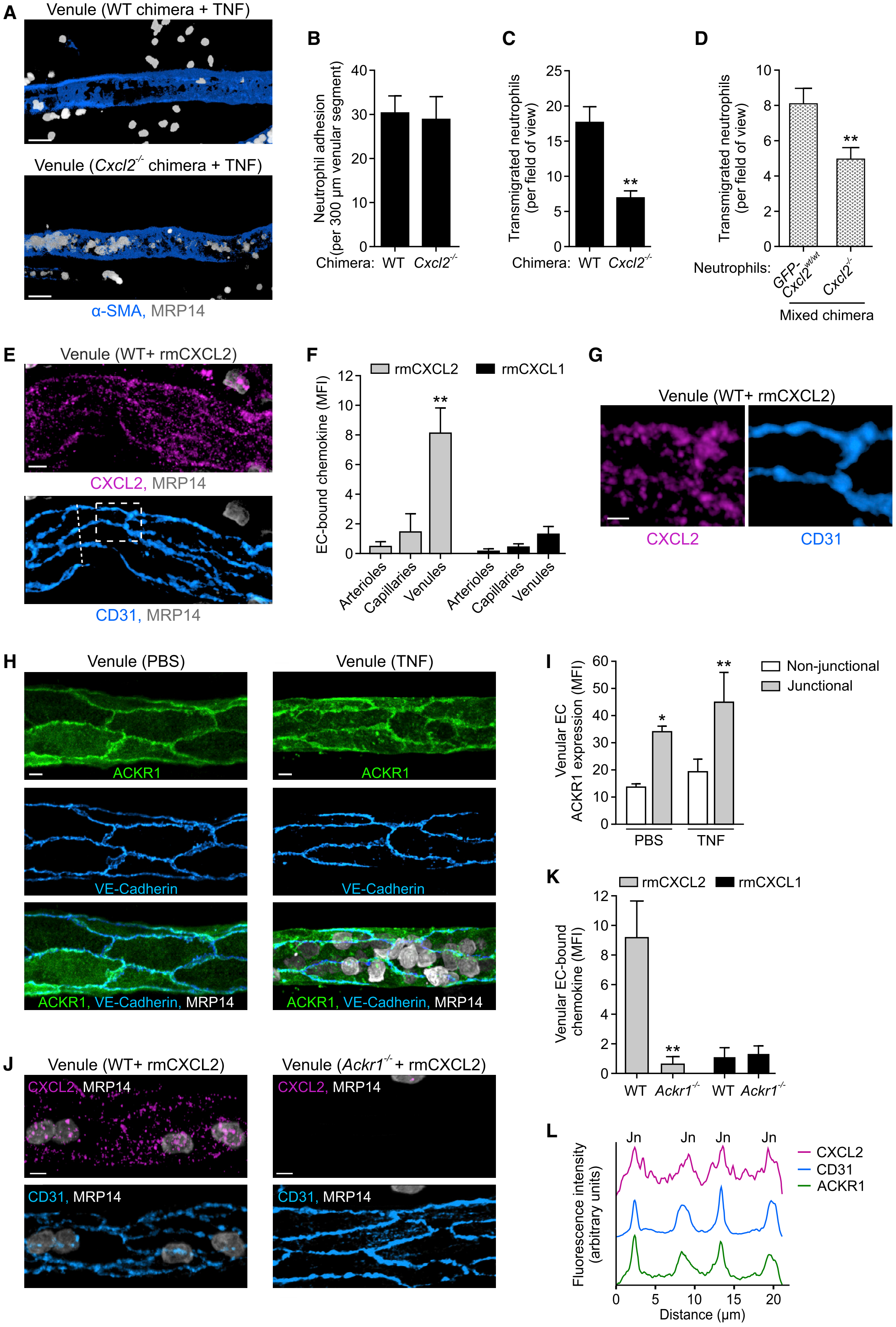

(legend on next page)

produced by ECs and pericytes and CXCL2 being expressed and secreted by stimulated neutrophils.

CXCL2 Mediates Neutrophil TEM in a Cell-Autonomous Manner and Is Retained at EC Junctions via the Atypical Chemokine Receptor ACKR1

As endogenous CXCL2 plays a vital role in supporting luminal-to-abluminal neutrophil TEM (Figures 2E–2H) and a rich source of this chemokine are TNF-stimulated neutrophils (Figures 5E–5H), we sought to investigate the role of neutrophil-derived CXCL2 in neutrophil transmigration. For this purpose, we generated leukocyte CXCL2-deficient (*Cxcl2*$^{-/-}$) and control (WT) chimeras by transferring bone marrow cells from *Cxcl2*$^{-/-}$ or WT mice, respectively, into lethally irradiated *Lyz2-EGFP-ki* recipients. Initial control studies demonstrated that CXCL2-expressing and *Cxcl2*$^{-/-}$ neutrophils show similar chemotaxis responses in an *in vitro* assay (Figure S5A). In TNF-stimulated cremaster muscles, *Cxcl2*$^{-/-}$ chimeras exhibited comparable levels of neutrophil adhesion but significantly reduced neutrophil extravasation, as compared to WT chimeras (Figures 6A–6C). Similarly, *Cxcl2*$^{-/-}$ chimeras showed reduced TNF-induced neutrophil (but not monocyte) extravasation into peritoneal cavities and decreased neutrophil infiltration in a lipopolysaccharide (LPS)-driven cutaneous inflammation model (Figures S5B–S5D). To gain greater insight into the mechanism through which neutrophil CXCL2 promotes neutrophil migration, we generated mixed chimeric mice that expressed both *Cxcl2*$^{-/-}$ and *GFP-Cxcl2*$^{wt/wt}$ neutrophils. Here, direct comparison of *Cxcl2*$^{-/-}$ and *GFP-Cxcl2*$^{wt/wt}$ neutrophils within TNF-stimulated cremaster muscles showed a significantly reduced transmigration response of the *Cxcl2*$^{-/-}$ cells (Figure 6D). Together, these findings provide direct evidence for the ability of neutrophil-derived CXCL2 to mediate neutrophil TEM and suggest that this response is, at least partly, mediated in a self-guided autocrine manner.

In aiming to ascertain whether the binding of neutrophil-derived CXCL2 to ECs was a component of its functionality, we investigated the binding profile of locally applied recombinant murine (rm)CXCL2 in cremaster muscles. IF staining of permeabilized tissues showed remarkably selective binding of rmCXCL2 to venular ECs with negligible binding to arteriolar and capillary ECs (Figures 6E, 6F, and S5E). Of note, rmCXCL2 binding was co-localized with CD31^{hi} regions, indicating enrichment at EC junctions (Figures 6E and 6G). In contrast, exogenous rmCXCL1 exhibited only marginal binding to venules and showed no indications of junctional enrichment (Figure 6F and S5E). As the junctional binding profile of rmCXCL2 is consistent with the functional role of CXCL2 in mediating neutrophil TEM, we sought to investigate the mechanism through which soluble CXCL2 is retained at EC junctions. Here, we focused our attention on the atypical chemokine receptor ACKR1 which binds CXCL2 and numerous other CXC and CC chemokines with high affinity (Nibbs and Graham, 2013; Novitzky-Basso and Rot, 2012). ACKR1 expression has recently been comprehensively studied in the murine microcirculation, identifying it as a specific marker of venular ECs in multiple tissues (Thiriot et al., 2017). We confirmed this in our system and showed additionally that irrespective of TNF treatment, ACKR1 expression was exclusively venular within the mouse cremaster muscle microcirculation (Figure S5F). Notably, ACKR1 expression was enriched at EC junctions (as identified by VE-Cadherin staining) and no change in the profile or magnitude of this expression was noted in inflamed tissues (Figures 6H and 6I). A junctional expression profile for ACKR1 was also observed in the ear dermal microcirculation of WT mice but not *Ackr1*$^{-/-}$ mice, confirming the specificity of our anti-ACKR1 mAb (Figure S5G). We next sought to investigate the possibility that ACKR1 may support EC junctional retention of CXCL2. Indeed, the venular binding of rmCXCL2 (but not rmCXCL1) was totally dependent on ACKR1 as indicated by its lack of binding in tissues of *Ackr1*$^{-/-}$ mice (Figures 6J and 6K). Detailed analysis of ACKR1 expression and the binding pattern of exogenous rmCXCL2 showed clear EC junctional association of both molecules as demonstrated by their overlap with EC CD31^{hi} regions (Figure 6L).

Together these data provide quantitative evidence for enriched ACKR1 expression at EC junctions and show that this expression pattern allows ACKR1 to retain non-EC-derived CXCL2 at EC junctions.

Endothelial ACKR1 Facilitates Decisive Luminal-to-Abluminal Neutrophil TEM

Next, we sought to investigate the functional role of EC ACKR1 in neutrophil TEM *in vivo*. Initial IF staining revealed that in TNF-stimulated venules, neutrophils breached EC junctions in close apposition to regions of high ACKR1 expression. Notably, while

Figure 6. CXCL2 Mediates Neutrophil TEM in a Cell-Autonomous Manner and Is Retained at EC Junctions via the Atypical Chemokine Receptor ACKR1

(A–C) Representative confocal images of venules in TNF-stimulated cremaster muscles of control (WT) or leukocyte CXCL2-deficient (*Cxcl2*$^{-/-}$) chimeras stained for α-SMA and MRP14 (A) and associated quantifications of neutrophil adhesion and extravasation (B, C; n = 4 mice per group) from 4 independent experiments). Scale bars, 30 μm.

(D) Numbers of extravasated *GFP-Cxcl2*$^{wt/wt}$ and *Cxcl2*$^{-/-}$ neutrophils in TNF-stimulated cremaster muscles of mixed chimeras, as normalized for neutrophil numbers in the blood (n = 4 mice, 3 independent experiments).

(E–G) RmCXCL2 or rmCXCL1 was injected i.s. into WT mice and cremaster muscles were stained for CXCL2 or CXCL1, CD31, and MRP14 and analyzed by confocal microscopy. Images (E; representative of 4 independent experiments; scale bar, 5 μm), quantified localization of rmCXCL2 or rmCXCL1 within the microcirculation (F, n = 3–8 mice per group, 8 independent experiments), and enlarged images of the boxed region in (E) (G; scale bar, 2 μm).

(H and I) Cremaster muscles were stimulated with PBS or TNF and IF stained for ACKR1, VE-Cadherin, and MRP14. Confocal images of venules (H; scale bars, 5 μm) and quantification of ACKR1 localization (I, n = 4 mice per group, 4 independent experiments).

(J–L) IF localization of rmCXCL2 and rmCXCL1 in cremasteric venules in relation to ACKR1 expression. Representative confocal images of tissues from WT and *Ackr1*$^{-/-}$ mice injected with rmCXCL2 (J; scale bars, 5 μm) and quantifications of endothelial rmCXCL2 and rmCXCL1 binding (K, n = 4–5 mice per group, 3 independent experiments). (L) CXCL2, ACKR1, and CD31 IF intensities along the dashed line in (E) cutting across 4 EC junctions (Jn; representative of 4 independent experiments). Means ± SEM, *p < 0.05, **p < 0.01 (as compared to arterioles or capillaries in F and non-junctional ACKR1 expression in I).

See also Figure S5.

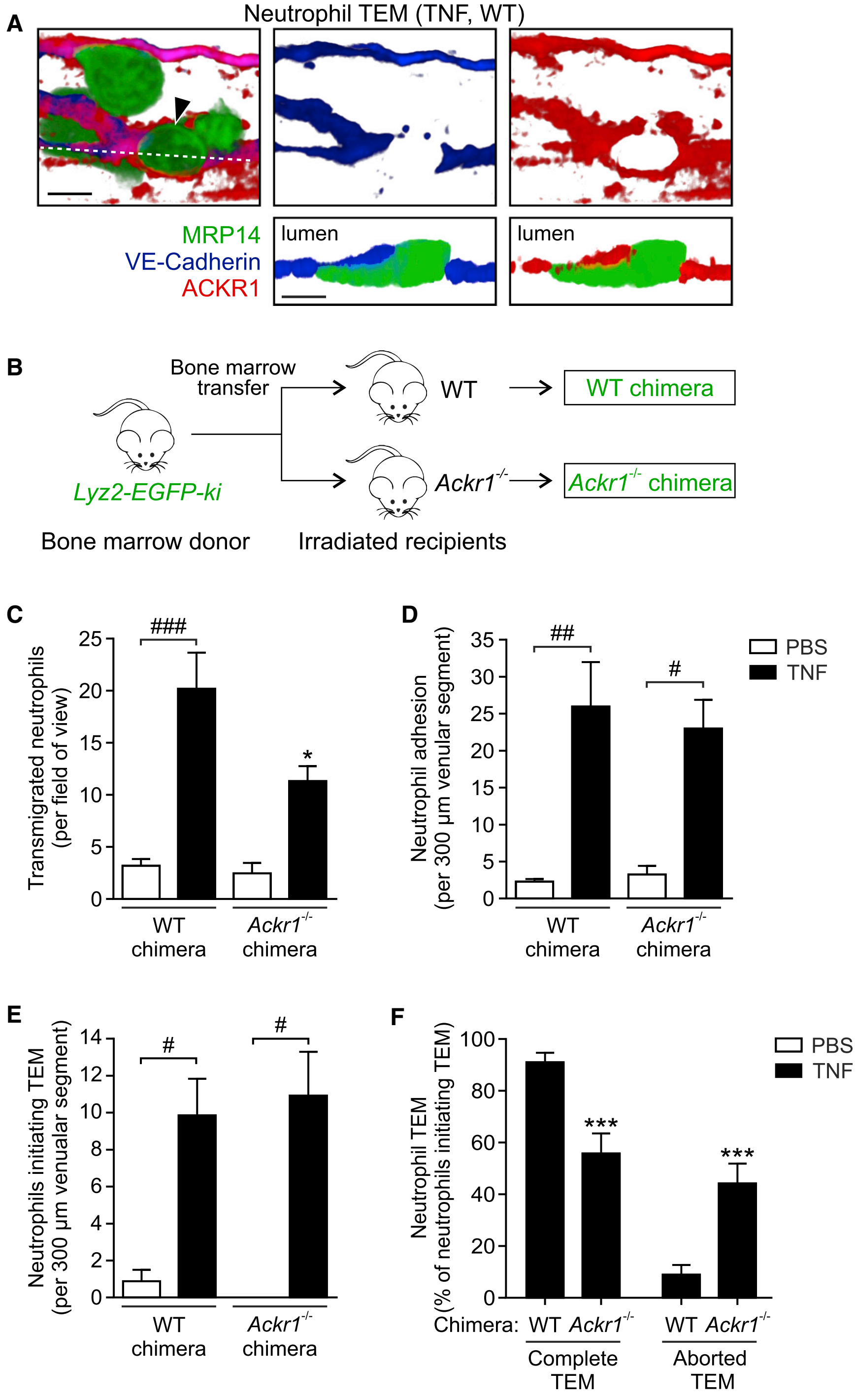

Figure 7. Endothelial ACKR1 Facilitates Decisive Luminal-to-Abluminal TEM

(A) Confocal images showing a neutrophil (arrow) migrating through an EC junction in a TNF-stimulated cremasteric venule of a WT mouse IF stained for MRP14, VE-Cadherin, and ACKR1. Luminal (top) and cross-sectional images (bottom) along dashed line; representative of 3 independent experiments; scale bars, 5 μm.

(B) Scheme illustrating the generation of chimeric mice exhibiting *Lyz2-EGFP-ki* hematopoietic cells (GFP^+ neutrophils) and WT or *Ackr1*$^{-/-}$ non-hematopoietic cells.

(C–F) Quantification of neutrophil extravasation (C), adhesion to ECs (D), initiating TEM (E), and undergoing complete or aborted TEM (F) in cremaster muscles of WT and *Ackr1*$^{-/-}$ chimeras after local PBS or TNF injection (n = 3–8 mice per group, 26 independent experiments) as analyzed by confocal IVM. Means ± SEM, *p < 0.05, ***p < 0.001 relative to WT chimeras and #p < 0.05, ##p < 0.01, ###p < 0.001 as indicated.

See also Figure S6.

VE-Cadherin was displaced during neutrophil TEM, ACKR1 formed ring-like structures around leading edge protrusions of transmigrating neutrophils (Figure 7A). This supports the notion that neutrophils may sense chemokines within EC junctions as immobilized by ACKR1. Unlike other ACKRs (Nibbs and Graham, 2013), ACKR1 does not scavenge its cognate chemokines but supports their functions, including facilitating chemokine-mediated leukocyte emigration (Pruenster et al., 2009). However, the mechanism by which EC ACKR1 achieves this has not been studied *in vivo*. To directly investigate the role of EC ACKR1, chimeric mice were generated by bone-marrow transfer from *Lyz2-EGFP-ki* mice to lethally irradiated control mice (WT chimeras) and *Ackr1*$^{-/-}$ mice (*Ackr1*$^{-/-}$ chimeras; deficient in EC ACKR1) (Figure 7B). We compared migratory responses of GFP^+ neutrophils in CD31-labeled inflamed cremaster muscles of these mice by confocal IVM. Total neutrophil extravasation was significantly reduced in TNF-stimulated tissues of mice deficient in EC ACKR1 (~48%; Figure 7C). This, however, was not associated with reduced luminal neutrophil adhesion, which was not significantly different between WT and *Ackr1*$^{-/-}$ chimeric animals (Figure 7D). Furthermore, analysis of neutrophil TEM revealed that the number of neutrophils extending a protrusion through CD31-labeled EC junctions and creating a notable pore, i.e., initiating TEM, was similar in WT and *Ackr1*$^{-/-}$ chimeras (Figure 7E). However, in EC ACKR1-deficient animals, >40% of neutrophils that entered EC junctions exhibited aborted TEM, reversed their migratory direction, and re-entered the blood circulation. In contrast, WT chimeras showed only <10% of such disrupted mode of TEM (Figure 7F). Similar to findings with TNF, *Ackr1*$^{-/-}$ chimeras treated intrascrotally with IL-1β showed no defect in neutrophil adhesion, but had reduced TEM and increased aborted TEM, as compared to WT chimeras (Figures S6A and S6B). Furthermore, *Ackr1*$^{-/-}$ chimeras exhibited reduced tissue infiltration of neutrophils in a TNF-driven peritonitis model

(Figure S6C) and in models of cutaneous inflammation as elicited by local injection of TNF or LPS (Figure S6D and S6E). Of note, TNF-induced monocyte migration into the peritoneal cavity was also decreased in *Ackr1*$^{-/-}$ chimeras (Figure S6F), suggesting a role for EC junctional ACKR1 in retention of monocyte recruiting chemokines (e.g., CCL2) and hence monocyte TEM.

Collectively these results identify a function for EC ACKR1 as a receptor pivotal for retaining extrinsic chemokines at EC junctions and as such supporting persistent directional neutrophil migration through EC junctions in a luminal-to-abluminal manner.

DISCUSSION

Despite our growing understanding of the cellular and molecular events that support leukocyte trafficking, details of the pro-migratory mechanisms that guide leukocytes through the complex bi-cellular and 3D structure of venular walls remains unclear. Here, we demonstrate that the passage of neutrophils from the bloodstream to the interstitial tissue is governed by the existence of sequentially expressed and molecularly distinct "chemotactic depots" that are presented to neutrophils on the luminal aspect of the endothelium, within EC junctions, and in the pericyte layer. These consecutive pro-migratory signals are established through cell-specific generation of defined chemokines and the existence of functionally pivotal retention mechanisms. The delineation of distinct cellular and molecular compartments within venular walls provides a paradigm for regulation of efficient and persistent neutrophil transmigration.

Although the scope of chemokine functions now extends well beyond their original characterization as leukocyte chemoattractants, chemokines remain among the most potent and versatile pro-migratory mediators of *in vivo* leukocyte migration and trafficking. With respect to neutrophils, the ELR^{+} CXC chemokines, human CXCL8 (IL-8), and its murine functional homologs, CXCL1 (KC) and CXCL2 (MIP-2), are among the most effective drivers of neutrophil migration into inflamed tissues (Griffith et al., 2014). The principal receptors for ELR^{+} CXC chemokines are CXCR1 and CXCR2, with the latter being of particular interest due to its direct association with numerous acute inflammatory pathologies (Stadtmann and Zarbock, 2012). The essential involvement of CXCR2 in supporting acute neutrophil trafficking is well established but the precise roles of its multiple ligands (i.e., CXCL1, CXCL2, CXCL3, CXCL5, CXCL7, and MIF) remain unclear with suggestions of functional overlap or redundancy. Here, within a CXCR2-dependent inflammatory model, we identified distinct, sequential, and functionally non-redundant roles for endogenously generated CXCL1 and CXCL2 in driving neutrophil migration through venular walls. Specifically, TNF-induced CXCL1 mediated neutrophil adhesion and intraluminal crawling on inflamed ECs and sub-EC crawling on pericytes, whereas CXCL2 supported neutrophil breaching of EC junctions. Mechanistically, this was attributed to the defined cellular sources of CXCL1 and CXCL2 in that TNF-activated ECs and pericytes selectively generated CXCL1, while CXCL2 was derived primarily from neutrophils. This disparate profile of CXCL1 and CXCL2 sources is in line with similar observations stemming from other inflammatory models indicating relatively restricted expression of CXCL1 to vascular and tissue-resident cells, and associating CXCL2, but not CXCL1, with activated neutrophils (Li et al., 2016). Nevertheless, CXCL1 and CXCL2 injected into tissues are often used interchangeably to induce neutrophil migration *in vivo*, and indeed both have been shown to activate all the steps of the leukocyte adhesion cascade (Zhang et al., 2001). However, in agreement with our findings, endogenously produced CXCL1 and CXCL2 can exhibit distinct functional profiles in different experimental models (Armstrong et al., 2004; Li et al., 2016; Chou et al., 2010), demonstrating the existence and indeed strength of sequential cascades of pro-inflammatory mediators in development of neutrophilic inflammation (Sadik et al., 2011).

The ability of EC- and pericyte-associated CXCL1 in supporting neutrophil crawling and adhesion is well in line with the essential role of CXCR2 in activating β_2-integrin inside-out signaling (Lefort and Ley, 2012). Mechanistically, both stimulated ECs and pericytes exhibit enhanced ICAM-1 expression (Proebstl et al., 2012) and as such neutrophil crawling on these cells is ICAM-1 dependent (Phillipson et al., 2006; Proebstl et al., 2012). Of note, the importance of pericyte-associated CXCL1 in maintaining persistent and unidirectional movement of neutrophils through venular walls was dramatically illustrated by the occurrence of neutrophil reverse motility within venular walls, and ultimately re-entry back into the vascular lumen, under conditions of CXCL1 blockade. We have previously associated such neutrophil reverse TEM (rTEM) with inflammatory conditions exhibiting neutrophil elastase (NE)-mediated cleavage of EC JAM-C (Colom et al., 2015; Woodfin et al., 2011). JAM-C expression is not, however, regulated during TNF-induced inflammation and both TNF and CXCL1 are not effective inducers of NE release from neutrophils (data not shown). Thus, altered expression of JAM-C does not account for the aberrant modes of neutrophil TEM noted in the present study. The CXCL1 dependency of luminal neutrophil responses and neutrophil-pericyte interactions may, however, be supported by the retention of EC- and pericyte-derived CXCL1 by cell surface glycosaminoglycans (GAGs) that characteristically retain cell-autonomous chemokines (Proudfoot et al., 2017).

Within the peripheral circulation, migration of leukocytes through the endothelium typically occurs via contacts between adjacent ECs as facilitated by numerous junctionally presented adhesion molecules (Nourshargh and Alon, 2014). Although our understanding of the expression and function of such molecules is growing (Reglero-Real et al., 2016), less is known about the patterning of directional cues that govern neutrophil protrusion through junctions and breaching of the endothelium. Here we show that local blockade of CXCL2, but not CXCL1, resulted in an aborted mode of neutrophil transit through EC junctions and reverse motility of neutrophils back into the vascular lumen. The use of chimeric mice and mixed chimeric mice deficient in leukocyte CXCL2 provided compelling evidence to suggest the establishment of a cell-autonomous neutrophil chemotaxis response within EC junctions. Neutrophils are known to be a rich source of chemokines that can be released by a broad range of stimuli *in vitro* (Tecchio and Cassatella, 2016) and indeed neutrophil-derived chemokines and other chemoattractants can act in a feed forward loop to support neutrophil migration and swarming in interstitial tissues, as well as the migration of other leukocyte sub-types into inflammatory sites (Chou et al.,

2010; Lämmermann et al., 2013; Li et al., 2016; Lim et al., 2015). In identifying a role for neutrophil-derived CXCL2 in neutrophil TEM, our results reveal a function of leukocyte-derived chemokines, and potentially other chemotactic agents, in facilitating immune cell breaching of EC junctions. Mechanistically, we noted that cell-extrinsic CXCL2 (but not CXCL1) selectively bound to venular ECs in a manner entirely dependent on the atypical chemokine receptor ACKR1. Members of this family are structurally similar to signaling chemokine G protein-coupled receptors (GPCRs), but while they are unable to trigger signaling pathways characterized by classical chemokine receptors, ACKRs play critical roles in retention, transport, and clearance of chemokines (Nibbs and Graham, 2013). ACKR1 can bind CXCL2 and >20 other chemokines and whereas its expression in erythroid lineage regulates hematopoiesis (Duchene et al., 2017) and availability of plasma chemokines (Novitzky-Basso and Rot, 2012), EC ACKR1 supports leukocyte trafficking (Pruenster et al., 2009). The latter was linked with the ability of EC ACKR1 to facilitate the internalization and transport of tissue-derived chemokines in a basolateral-to-apical manner across the EC barrier, triggering leukocyte adhesion and transmigration through the endothelium (Pruenster et al., 2009). Such studies involved using exogenous chemokines applied to either unstimulated tissues or cultured ECs *in vitro* and under conditions of ACKR1 overexpression. However, the role of constitutively expressed ACKR1 in mediating different stages of neutrophil extravasation *in vivo*, and most importantly as elicited by endogenous chemokines, has not been addressed before. A role for ACKR1 in regulation of neutrophil migration is well in line with its selective expression on venular ECs (Thiriot et al., 2017), but its EC junctional expression and ability to retain CXCL2, together with defective neutrophil TEM in EC ACKR1-deficient mice, revealed a role for ACKR1 in regulation of neutrophil TEM. Although we cannot rule out potential contribution of GAG-bound CXCL2 to ACKR1-dependent and -independent neutrophil migration through EC junctions, our results suggest that at sites of inflammation, neutrophils require an EC junctional ACKR1-dependent depot of neutrophil-derived CXCL2 that facilitates their directional migration through EC junctions. Of note, we also found that exogenous CXCL2 can be endocytosed and localized in discrete vesicles in ECs (in addition to its enrichment within EC junctions), which is consistent with the previously described role of ACKR1 in chemokine transcytosis (Minten et al., 2014; Pruenster et al., 2009). This indicates that chemokine endocytosis and junctional retention are not mutually exclusive and might occur simultaneously as governed by the nature of the inflammatory scenario.

Collectively, the present findings offer a diapedesis model whereby within inflamed venules, ECs and pericytes produce CXCL1 that is presented to migrating neutrophils on GAG scaffolds, thus supporting both luminal and sub-luminal neutrophil adhesion and crawling responses. With respect to breaching of the endothelium, we propose that neutrophils that squeeze through EC junctions secrete CXCL2, which locally binds and is retained by EC junctional ACKR1. We believe that the ACKR1-CXCL2 axis provides a potential mechanism whereby a localized and temporally regulated chemotactic depot supports TEM through local guidance in an autocrine manner. Our findings suggest that such compartmentalization of CXCL1 and CXCL2, and orderly presentation to neutrophils, is critical in maintaining continued and persistent migration of neutrophils through venular walls via the same GPCR. Furthermore, while released chemokines are prone to proteolytic degradation, neutrophil-derived CXCL2 within the confined region of EC junctions might remain protected from both proteolytic inactivation and dilution by blood flow. The establishment of an EC junctional CXCL2 deposition, as mediated via junctional ACKR1, as a mechanism for supporting neutrophil TEM is reminiscent of the role of localized EC intracellular CCL2 vesicular depots for supporting lymphocyte TEM (Shulman et al., 2011) and release of CCL21 at lymphatic EC junctions in supporting dendritic cell (DC) TEM (Vaahtomeri et al., 2017).

The chemokine superfamily exhibits many examples of promiscuity, with most chemokine receptors having several chemokine ligands and many ligands being shared by multiple receptors. In many scenarios this is believed to offer considerable flexibility and redundancy to the system, but there are also examples where multiple ligands act co-operatively to support key biological responses. One such scenario is offered by CCR7 and its ligands CCL19 and CCL21, which show distinct but co-operative roles in promoting DC migration to secondary lymphoid tissues (Schumann et al., 2010; Weber et al., 2013). With respect to CXCR2, although full details of the functional roles of its multiple ligands are unclear, there is emerging evidence for divergent properties. For example, CXCL1 and CXCL2 exhibit differential GAG binding dynamics (Tanino et al., 2010) and human CXCR2 exhibits biased signaling upon ligand binding (Rajagopal et al., 2013). While these functions suggest differing biological profiles, the associated mechanisms and relevance during inflammatory responses *in vivo* remain unclear. Our results provide evidence for the existence of distinct but supportive roles for the CXCR2 ligands CXCL1 and CXCL2 and show that these properties are tightly regulated by both cell-intrinsic and -extrinsic factors that cooperate in guiding neutrophils through inflamed venular walls. Collectively, these results support the emerging concept that in inflammatory conditions, functional gradients are subjected to temporal regulation of chemokines that are locally generated and strategically presented to migrating cells by specialized cellular and extracellular scaffolds. As such, increased understanding of temporal and localized generation of inflammatory mediators in distinct disease models could identify more efficacious and selective modes of targeting pathological immune cell trafficking.

STAR★METHODS

Detailed methods are provided in the online version of this paper and include the following:

- KEY RESOURCES TABLE
- CONTACT FOR REAGENT AND RESOURCE SHARING
- EXPERIMENTAL MODEL AND SUBJECT DETAILS
- METHOD DETAILS
    - Inflammatory response in cremaster muscles
    - Whole-mount IF staining
    - Confocal microscopy and image analysis
    - Peritoneal inflammation
    - Flow cytometry

- ○ Confocal intravital microscopy (IVM)
- ○ Neutrophil isolation
- ○ Ca^{2+} flux assay
- ○ *In vitro* neutrophil adhesion assay
- ○ Western Blot
- ○ Transwell chemotaxis assay
- ○ Fluorescence *in situ* hybridization (FISH)
- ○ Pericyte isolation and culture
- ○ Real-time PCR
- ○ *In vitro* pericyte and neutrophil analysis
- ○ Generation of bone marrow chimeric mice
- ○ Dorsal skin inflammation
- ○ *In vivo* rmCXCL2 and rmCXCL1 binding assay
- ● QUANTIFICATION AND STATISTICAL ANALYSES

SUPPLEMENTAL INFORMATION

Supplemental Information includes six figures and four videos and can be found with this article online at https://doi.org/10.1016/j.immuni.2018.09.018.

ACKNOWLEDGMENTS

We thank Dr. Christian Weber for providing the anti-MIF mAb. This work was supported by funds from the British Heart Foundation (FS/14/3/30518 to T.G. and S.N.), the People Programme (Marie Curie Actions) of the EU's 7th Framework Programme (FP7/2007-2013) under REA grant agreement 608765 (to T.G. and S.N.), and by the Wellcome Trust (098291/Z/12/Z to S.N.). D.S. is supported by the CNIC, SAF2016-79040-R from the Spanish Ministerio de Ciencia, and ERC-2016-CoG 725091 from the European Research Council. M.T. and A.R. are supported by the Sinergia grant CRSII3_160719 of the Swiss National Science Foundation. G.G. is supported by the Wellcome Trust and the MRC. U.H.v.A. and A.T. are supported by the Ragon Institute of MGH, MIT and Harvard and the HMS Center for Immune Imaging.

AUTHOR CONTRIBUTIONS

Conceptualization, writing (original draft), and funding acquisition, T.G., S.N.; Methodology, T.G., M.-B.V., S.N.; Investigation and formal analysis, T.G., T.L., L.P., L.R., A.B., E.L., E.H.; Resources, A.T., C.F., M.T., G.G., D.S., U.H.v.A., A.R.; Supervision, R.A., M.-B.V., A.R., S.N.; Writing (review and editing), all authors.

DECLARATION OF INTERESTS

The authors declare no competing interests.

Received: March 20, 2018
Revised: August 13, 2018
Accepted: September 21, 2018
Published: November 13, 2018

REFERENCES

Armstrong, D.A., Major, J.A., Chudyk, A., and Hamilton, T.A. (2004). Neutrophil chemoattractant genes KC and MIP-2 are expressed in different cell populations at sites of surgical injury. J. Leukoc. Biol. *75*, 641–648.

Cacalano, G., Lee, J., Kikly, K., Ryan, A.M., Pitts-Meek, S., Hultgren, B., Wood, W.I., and Moore, M.W. (1994). Neutrophil and B cell expansion in mice that lack the murine IL-8 receptor homolog. Science *265*, 682–684.

Chou, R.C., Kim, N.D., Sadik, C.D., Seung, E., Lan, Y., Byrne, M.H., Haribabu, B., Iwakura, Y., and Luster, A.D. (2010). Lipid-cytokine-chemokine cascade drives neutrophil recruitment in a murine model of inflammatory arthritis. Immunity *33*, 266–278.

Colom, B., Bodkin, J.V., Beyrau, M., Woodfin, A., Ody, C., Rourke, C., Chavakis, T., Brohi, K., Imhof, B.A., and Nourshargh, S. (2015). Leukotriene B4-neutrophil elastase axis drives neutrophil reverse transendothelial cell migration in vivo. Immunity *42*, 1075–1086.

Dawson, T.C., Lentsch, A.B., Wang, Z., Cowhig, J.E., Rot, A., Maeda, N., and Peiper, S.C. (2000). Exaggerated response to endotoxin in mice lacking the Duffy antigen/receptor for chemokines (DARC). Blood *96*, 1681–1684.

Del Fresno, C., Saz-Leal, P., Enamorado, M., Wculek, S.K., Martínez-Cano, S., Blanco-Menéndez, N., Schulz, O., Gallizioli, M., Miró-Mur, F., Cano, E., et al. (2018). DNGR-1 in dendritic cells limits tissue damage by dampening neutrophil recruitment. Science *362*, 351–356.

Duchene, J., Novitzky-Basso, I., Thiriot, A., Casanova-Acebes, M., Bianchini, M., Etheridge, S.L., Hub, E., Nitz, K., Artinger, K., Eller, K., et al. (2017). Atypical chemokine receptor 1 on nucleated erythroid cells regulates hematopoiesis. Nat. Immunol. *18*, 753–761.

Faust, N., Varas, F., Kelly, L.M., Heck, S., and Graf, T. (2000). Insertion of enhanced green fluorescent protein into the lysozyme gene creates mice with green fluorescent granulocytes and macrophages. Blood *96*, 719–726.

Griffith, J.W., Sokol, C.L., and Luster, A.D. (2014). Chemokines and chemokine receptors: positioning cells for host defense and immunity. Annu. Rev. Immunol. *32*, 659–702.

Hobbs, J.A., May, R., Tanousis, K., McNeill, E., Mathies, M., Gebhardt, C., Henderson, R., Robinson, M.J., and Hogg, N. (2003). Myeloid cell function in MRP-14 (S100A9) null mice. Mol. Cell. Biol. *23*, 2564–2576.

Kienle, K., and Lämmermann, T. (2016). Neutrophil swarming: an essential process of the neutrophil tissue response. Immunol. Rev. *273*, 76–93.

Lämmermann, T., Afonso, P.V., Angermann, B.R., Wang, J.M., Kastenmüller, W., Parent, C.A., and Germain, R.N. (2013). Neutrophil swarms require LTB4 and integrins at sites of cell death in vivo. Nature *498*, 371–375.

Lefort, C.T., and Ley, K. (2012). Neutrophil arrest by LFA-1 activation. Front. Immunol. *3*, 157.

Li, J.L., Lim, C.H., Tay, F.W., Goh, C.C., Devi, S., Malleret, B., Lee, B., Bakocevic, N., Chong, S.Z., Evrard, M., et al. (2016). Neutrophils self-regulate immune complex-mediated cutaneous inflammation through CXCL2. J. Invest. Dermatol. *136*, 416–424.

Lim, K., Hyun, Y.M., Lambert-Emo, K., Capece, T., Bae, S., Miller, R., Topham, D.J., and Kim, M. (2015). Neutrophil trails guide influenza-specific $CD8^+$ T cells in the airways. Science *349*, aaa4352.

Minten, C., Alt, C., Gentner, M., Frei, E., Deutsch, U., Lyck, R., Schaeren-Wiemers, N., Rot, A., and Engelhardt, B. (2014). DARC shuttles inflammatory chemokines across the blood-brain barrier during autoimmune central nervous system inflammation. Brain *137*, 1454–1469.

Nibbs, R.J., and Graham, G.J. (2013). Immune regulation by atypical chemokine receptors. Nat. Rev. Immunol. *13*, 815–829.

Nourshargh, S., and Alon, R. (2014). Leukocyte migration into inflamed tissues. Immunity *41*, 694–707.

Nourshargh, S., Hordijk, P.L., and Sixt, M. (2010). Breaching multiple barriers: leukocyte motility through venular walls and the interstitium. Nat. Rev. Mol. Cell Biol. *11*, 366–378.

Novitzky-Basso, I., and Rot, A. (2012). Duffy antigen receptor for chemokines and its involvement in patterning and control of inflammatory chemokines. Front. Immunol. *3*, 266.

Phillipson, M., Heit, B., Colarusso, P., Liu, L., Ballantyne, C.M., and Kubes, P. (2006). Intraluminal crawling of neutrophils to emigration sites: a molecularly distinct process from adhesion in the recruitment cascade. J. Exp. Med. *203*, 2569–2575.

Proebstl, D., Voisin, M.B., Woodfin, A., Whiteford, J., D'Acquisto, F., Jones, G.E., Rowe, D., and Nourshargh, S. (2012). Pericytes support neutrophil subendothelial cell crawling and breaching of venular walls in vivo. J. Exp. Med. *209*, 1219–1234.

Proudfoot, A.E.I., Johnson, Z., Bonvin, P., and Handel, T.M. (2017). Glycosaminoglycan interactions with chemokines add complexity to a complex system. Pharmaceuticals (Basel) *10*, 10.

Pruenster, M., Mudde, L., Bombosi, P., Dimitrova, S., Zsak, M., Middleton, J., Richmond, A., Graham, G.J., Segerer, S., Nibbs, R.J., and Rot, A. (2009). The

Duffy antigen receptor for chemokines transports chemokines and supports their promigratory activity. Nat. Immunol. *10*, 101–108.

Rajagopal, S., Bassoni, D.L., Campbell, J.J., Gerard, N.P., Gerard, C., and Wehrman, T.S. (2013). Biased agonism as a mechanism for differential signaling by chemokine receptors. J. Biol. Chem. *288*, 35039–35048.

Reglero-Real, N., Colom, B., Bodkin, J.V., and Nourshargh, S. (2016). Endothelial cell junctional adhesion molecules: role and regulation of expression in inflammation. Arterioscler. Thromb. Vasc. Biol. *36*, 2048–2057.

Sadik, C.D., Kim, N.D., and Luster, A.D. (2011). Neutrophils cascading their way to inflammation. Trends Immunol. *32*, 452–460.

Schumann, K., Lämmermann, T., Bruckner, M., Legler, D.F., Polleux, J., Spatz, J.P., Schuler, G., Förster, R., Lutz, M.B., Sorokin, L., and Sixt, M. (2010). Immobilized chemokine fields and soluble chemokine gradients cooperatively shape migration patterns of dendritic cells. Immunity *32*, 703–713.

Shulman, Z., Cohen, S.J., Roediger, B., Kalchenko, V., Jain, R., Grabovsky, V., Klein, E., Shinder, V., Stoler-Barak, L., Feigelson, S.W., et al. (2011). Transendothelial migration of lymphocytes mediated by intraendothelial vesicle stores rather than by extracellular chemokine depots. Nat. Immunol. *13*, 67–76.

Stadtmann, A., and Zarbock, A. (2012). CXCR2: From bench to bedside. Front. Immunol. *3*, 263.

Tanino, Y., Coombe, D.R., Gill, S.E., Kett, W.C., Kajikawa, O., Proudfoot, A.E., Wells, T.N., Parks, W.C., Wight, T.N., Martin, T.R., and Frevert, C.W. (2010). Kinetics of chemokine-glycosaminoglycan interactions control neutrophil migration into the airspaces of the lungs. J. Immunol. *184*, 2677–2685.

Tecchio, C., and Cassatella, M.A. (2016). Neutrophil-derived chemokines on the road to immunity. Semin. Immunol. *28*, 119–128.

Thiriot, A., Perdomo, C., Cheng, G., Novitzky-Basso, I., McArdle, S., Kishimoto, J.K., Barreiro, O., Mazo, I., Triboulet, R., Ley, K., et al. (2017). Differential DARC/ACKR1 expression distinguishes venular from non-venular endothelial cells in murine tissues. BMC Biol. *15*, 45.

Vaahtomeri, K., Brown, M., Hauschild, R., De Vries, I., Leithner, A.F., Mehling, M., Kaufmann, W.A., and Sixt, M. (2017). Locally triggered release of the chemokine CCL21 promotes dendritic cell transmigration across lymphatic endothelia. Cell Rep. *19*, 902–909.

Wang, S., Voisin, M.B., Larbi, K.Y., Dangerfield, J., Scheiermann, C., Tran, M., Maxwell, P.H., Sorokin, L., and Nourshargh, S. (2006). Venular basement membranes contain specific matrix protein low expression regions that act as exit points for emigrating neutrophils. J. Exp. Med. *203*, 1519–1532.

Weber, M., Hauschild, R., Schwarz, J., Moussion, C., de Vries, I., Legler, D.F., Luther, S.A., Bollenbach, T., and Sixt, M. (2013). Interstitial dendritic cell guidance by haptotactic chemokine gradients. Science *339*, 328–332.

Woodfin, A., Voisin, M.B., Beyrau, M., Colom, B., Caille, D., Diapouli, F.M., Nash, G.B., Chavakis, T., Albelda, S.M., Rainger, G.E., et al. (2011). The junctional adhesion molecule JAM-C regulates polarized transendothelial migration of neutrophils in vivo. Nat. Immunol. *12*, 761–769.

Zhang, X.W., Liu, Q., Wang, Y., and Thorlacius, H. (2001). CXC chemokines, MIP-2 and KC, induce P-selectin-dependent neutrophil rolling and extravascular migration in vivo. Br. J. Pharmacol. *133*, 413–421.

Zlotnik, A., and Yoshie, O. (2012). The chemokine superfamily revisited. Immunity *36*, 705–716.

STAR★METHODS

KEY RESOURCES TABLE

REAGENT or RESOURCE	SOURCE	IDENTIFIER
Antibodies		
Anti-mouse MRP14 (clone 2B10)	Gift from Dr N. Hogg (Cancer Research UK UK) (Hobbs et al., 2003)	NA
Anti-mouse α-SMA (clone 1A4)	Sigma-Aldrich	Cat#A5228; RRID: AB_262054
Anti-mouse CD31 (clone 390)	Thermo Fisher Scientific	Cat#16-0311-85; RRID: AB_468933
Anti-mouse VE-Cadherin (clone eBioBV14)	Thermo Fisher Scientific	Cat#14-1442-85; RRID: AB_891373
Anti-mouse ACKR1 (clone 6B7)	(Thiriot et al., 2017)	NA
Anti-mouse PDGFR-β (polyclonal)	R&D Systems	Cat#AF1042; RRID: AB_2162633
Anti-mouse NG2 (polyclonal)	Millipore	Cat#AB5320; RRID: AB_91789
Blocking anti-mouse CXCL1 (clone 48415)	R&D Systems	Cat#MAB453; RRID: AB_2087696
Blocking anti-mouse CXCL2 (clone 40605)	R&D Systems	Cat#MAB452; RRID: AB_2230058
Anti-mouse CXCL1 (polyclonal)	R&D Systems	Cat#AF-453-NA; RRID: AB_354495
Anti-mouse CXCL2 (polyclonal)	R&D Systems	Cat#AF-452-NA; RRID: AB_2086326
Blocking anti-mouse CXCL5 (clone 61905)	R&D Systems	Cat#MAB433; RRID: AB_2086587
Blocking anti-mouse MIF (clone CT 1C10)	Gift from Dr C. Weber (Ludwig Maximilians University Munich, Germany)	NA
Blocking anti-mouse CXCR2 (clone 242216)	R&D Systems	Cat#MAB2164; RRID: AB_358062
Alexa Fluor 647 anti-mouse CXCR2 (clone SA044G4)	Biolegend	Cat#149306; RRID: AB_2565694
Pacific Blue anti-mouse CD45 (clone 30-F11)	Biolegend	Cat#103126; RRID: AB_493535
Alexa Fluor 647 anti-mouse Ly6G (clone 1A8)	Biolegend	Cat#127610; RRID: AB_1134159
PE anti-mouse CD115 (clone AFS98)	Thermo Fisher Scientific	Cat#12-1152-83: RRID: AB_465809
Anti-pan-AKT (polyclonal)	Cell Signaling Technology	Cat#9272; RRID: AB_329827
Anti-phospho-AKT (clone D9E)	Cell Signaling Technology	Cat#4060; RRID: AB_2315049
Chemicals, Peptides, and Recombinant Proteins		
BSA, low endotoxin	Sigma-Aldrich	Cat#A9543
Bovine collagen type I	Advanced Biomatrix	Cat#5005
DMEM, low glucose	Thermo Fisher Scientific	Cat#11885084
Fluo-4, AM, cell permeant	Thermo Fisher Scientific	Cat#F14201
HALT protease and phosphatase inhibitor cocktail	Thermo Fisher Scientific	Cat#78440
LPS	Sigma-Aldrich	Cat#L4391
LTB_4	Cayman Chemical	Cat#20110
Pluronic F-127	Thermo Fisher Scientific	Cat#P3000MP
Probenecid, water soluble	Thermo Fisher Scientific	Cat#P36400
Recombinant human PEDF	Sigma-Aldrich	Cat#SRP4988
Recombinant murine CXCL1	Preprotech	Cat#250-11
Recombinant murine CXCL2	Preprotech	Cat#250-15
Recombinant human ICAM-1	R&D Systems	Cat#ADP4-050
Recombinant murine IL-1β	R&D Systems	Cat#401-ML-005/CF
Recombinant murine TNF-α aa 80-235	R&D Systems	Cat#410-MT-010/CF
3,3′,5,5′-tetramethylbenzidine substrate solution	Thermo Fisher Scientific	Cat#00-4201-56
Critical Commercial Assays		
Alexa Fluor 488 antibody labeling kit	Thermo Fisher Scientific	Cat# A20181
Alexa Fluor 555 antibody labeling kit	Thermo Fisher Scientific	Cat#A20187

(*Continued on next page*)

Continued		
REAGENT or RESOURCE	SOURCE	IDENTIFIER
Alexa Fluor 647 antibody labeling kit	Thermo Fisher Scientific	Cat#A20186
DyLight 405 antibody labeling kit	Thermo Fisher Scientific	Cat#53021
iQ SYBR Green supermix	Biorad	Cat#1708880
Neutrophil isolation kit (mouse)	Miltenyi Biotec	Cat#130-097-658
Quantikine ELISA mouse CXCL1	R&D Systems	Cat#MKC00B
Quantikine ELISA mouse CXCL2	R&D Systems	Cat#MM200
RNAscope fluorescent multiplex assay	Advanced Cell Diagnostics	Cat#320851
Experimental Models: Organisms/Strains		
Mouse, *Ackr1*$^{-/-}$	(Dawson et al., 2000)	NA
Mouse, *Acta2-RFPcherry-Tg*	Gift from Dr D. Rowe (University of Connecticut Health Center, US) (Proebstl et al., 2012)	NA
Mouse, *Lyz2-EGFP-ki;Acta2-RFPcherry-Tg*	(Proebstl et al., 2012)	NA
Mouse, *Cxcl2*$^{-/-}$	The Jackson Laboratory	JAX 029557
Mouse, *Cxcr2*$^{-/-}$	(Cacalano et al., 1994)	NA
Mouse, C57BL/6	Charles River Laboratories	JAX 000664
Mouse, *Lyz2-EGFP-ki*	Gift from Dr M. Sperandio (Ludwig Maximilians University Munich, Germany) (Faust et al., 2000)	NA
Oligonucleotides		
mRNA probe: *Pecam1*-C3	Advanced Cell Diagnostics	Cat#316721-C3
mRNA probe: *Acta2*-C2	Advanced Cell Diagnostics	Cat#319531-C2
mRNA probe: *Cxcl1*-C1	Advanced Cell Diagnostics	Cat#407721
mRNA probe: *Cxcl2*-C1	Advanced Cell Diagnostics	Cat#437581
Real-time PCR primer: *Gapdh* forward 5′-TCGTGGATCTGACGTGCCGCCTG-3′	This paper	NA
Real-time PCR primer: *Gapdh* reverse 5′-CACCACCCTGTTGCTGTAGCCGTA-3′	This paper	NA
Real-time PCR primer: *Cxcl1* forward 5′-CCGAAGTCATAGCCACACTCAA-3′	This paper	NA
Real-time PCR primer: *Cxcl1* reverse 5′-GCAGTCTGTCTTCTTTCTCCGTTA-3′	This paper	NA
Real-time PCR primer: *Cxcl2* forward 5′-GAAGTCATAGCCACTCTCAAGG-3′	This paper	NA
Real-time PCR primer: *Cxcl2* reverse 5′-CCTCCTTTCCAGGTCAGTTAGC-3′	This paper	NA
Software and Algorithms		
Chemotaxis and migration tool v2.0	IBIDI	https://ibidi.com/manual-image-analysis/171-chemotaxis-and-migration-tool.html
FlowJo v10.2	Tree Star	https://www.flowjo.com/
ImageJ 1.49	NIH	https://imagej.nih.gov/ij/
Imaris v8	Bitplane	http://www.bitplane.com/imaris
Prism v6.05	GraphPad	https://www.graphpad.com/scientific-software/prism/
Other		
Corning Transwell inserts	Sigma-Aldrich	Cat#CLS3415-48EA
123count eBeads™ counting beads	Thermo Fisher Scientific	Cat#01-1234-42

CONTACT FOR REAGENT AND RESOURCE SHARING

Further information and requests for resources and reagents should be directed to and will be fulfilled by the Lead Contact, Sussan Nourshargh (s.nourshargh@qmul.ac.uk). The supply of the following reagents and mice are subject to MTA agreements with the

academics indicated in parenthesis: Anti-ACKR1 mAb (Dr Ulrich H von Andrian), *Lyz2-EGFP-ki* mice (Dr Thomas Graf) and *Acta2-RFPcherry-Tg* mice (Dr David Rowe).

EXPERIMENTAL MODEL AND SUBJECT DETAILS

WT C57BL/6 mice were purchased from Charles River Laboratories, UK. The *Lyz2-EGFP-ki* mouse colony was kindly provided by Dr Markus Sperandio (Ludwig Maximilians University of Munich, Germany) and used with the permission of Dr Thomas Graf (Center for Genomic Regulation and ICREA, Spain). These mice contain an EGFP gene that was knocked into the lysozyme M (*Lyz2*) locus to generate GFP^{+} myeloid cells (GFP^{bright} neutrophils, GFP^{dim} monocytes and macrophages) and were backcrossed with C57BL/6 mice for at least 8 generations (Faust et al., 2000). *Acta2-RFPcherry-Tg* mice were previously generated on a C57BL/6 background (Proebstl et al., 2012) and contain a transgenic insertion of the RFP variant cherry under control of the *Acta2* promotor and express RFP^{+} pericytes and smooth muscle cells. The *Lyz2-EGFP-ki*;*Acta2-RFPcherry-Tg* colony was obtained by crossing the *Lyz2-EGFP-ki* colony with the *Acta2-RFPcherry-Tg* colony (Proebstl et al., 2012). *Ackr1*$^{-/-}$ mice (Dawson et al., 2000) and *Cxcr2*$^{-/-}$ mice (Cacalano et al., 1994) were backcrossed onto a C57BL/6 background for at least 11 generations. *Cxcr2*$^{-/-}$ and WT mice exhibited similar levels of circulating neutrophils as determined 4 hr after i.s. TNF injection (1135 and 1723 neutrophils/μl blood respectively, $p > 0.5$, $n = 7\text{-}8$ mice per group). The *Cxcl2*$^{-/-}$ mice on C57BL/6 background were generated from heterozygotes from The Jackson Laboratory and were bred at the CNIC under specific pathogen-free (SPF) conditions (Del Fresno et al., 2018). All animals were group housed in individually ventilated cages under SPF conditions at the William Harvey Research Institute, Queen Mary University of London, UK. Male mice were used for studying responses in the cremaster muscle and dorsal skin and female mice were used for peritonitis experiments and analyses of the dermal ear vasculature. All experiments were carried out using 8-12-week-old mice (age and sex matched groups) and were performed in accordance with the UK Home Office legislation.

METHOD DETAILS

Inflammatory response in cremaster muscles

Mice were anaesthetized with 3% isoflurane and injected i.s. with 300 ng TNF or 50 ng IL-1β (both R&D Systems), whereas control mice received 400 μl PBS (2-4 hr incubation). For the analysis of total neutrophil extravasation blocking anti-CXCL1, anti-CXCL2, anti-CXCL5 (all R&D Systems) or anti-MIF mAbs (kindly provided by Dr Christian Weber, Ludwig Maximilians University of Munich, Germany) or corresponding isotype control mAbs (30 μg/mouse, R&D Systems) were injected i.s. together with TNF. For IVM analyses mAbs were applied as described in the corresponding section below.

Whole-mount IF staining

Cremaster muscles or ears were fixed in 4% paraformaldehyde (PFA, Sigma-Aldrich) for 1 hr at 4°C and permeabilized and blocked in PBS containing 0.5% Triton X-100 (Sigma-Aldrich) and 25% fetal calf serum (FCS, Thermo Fisher Scientific) for 4 hr at room temperature. Subsequently, the tissues were incubated with unlabeled or fluorescently labeled primary antibodies in PBS containing 10% FCS over night at 4°C. Where required, samples were incubated with fluorescently labeled secondary antibodies in PBS containing 10% FCS for 3 hr at room temperature. Antibody conjugation to Alexa Fluor 488, 555, 647 or DyLight 405 fluorophores was carried out using labeling kits (Thermo Fisher Scientific) according to the manufacturer's recommendations.

Confocal microscopy and image analysis

Immunostained whole-mount cremaster muscles or ears were imaged with an up-right Leica TCS SP5 (Leica) or inverted Zeiss 800 (Carl Zeiss) confocal laser scanning microscope equipped with argon and helium lasers (488, 561 and 633 nm excitation wavelengths) or solid-state laser diodes (405, 488, 561 and 640 nm excitation wavelengths), respectively. Serial z stacks of postcapillary venules (diameter 20-45 μm) were acquired with a water dipping 20x (1 NA) objective or oil immersion 40x (1.3 NA) or 63x (1.4 NA) objectives and the resulting images of half vessels were reconstructed in 3D and analyzed using Imaris software (Bitplane). Neutrophil extravasation in cremaster muscles was determined by immunostaining using mAbs against MRP14 (kindly provided by Dr Nancy Hogg, Cancer Research UK, UK), CD31 (Thermo Fisher Scientific) and α-SMA (Sigma-Aldrich) to label neutrophils, ECs and pericytes, respectively. Extravasated neutrophils per field of view (330 × 160 × 45 μm) were quantified from 8-10 images per mouse. *Cxcl2*$^{-/-}$ and *GFP-Cxcl2*$^{wt/wt}$ neutrophils in mixed chimeric mice were identified based on their $MRP14^{+}/GFP^{-}$ and $MRP14^{+}/GFP^{+}$ expression profile, respectively, and normalized for neutrophil numbers in the blood. Adherent neutrophils in *Cxcl2*$^{-/-}$ chimeras were determined by IF staining and defined as luminal neutrophils attached to ECs. Endogenous chemokine expression or rmCXCL1 or rmCXCL2 binding within venular ECs and pericytes was analyzed using polyclonal anti-CXCL1 or anti-CXCL2 antibodies (R&D Systems) as described (Woodfin et al., 2011). Briefly, EC and pericyte isosurfaces were created based on regions immunostained for CD31 (including $CD31^{high}$ junctional and $CD31^{dim}$ non-junctional regions) or α-SMA, respectively, and chemokine MFI values per unit area within these isosurfaces were determined. The pericyte marker expression profile was established using anti-α-SMA, anti-PDGFR-β (R&D Systems), and anti-NG2 (Millipore) antibodies. ACKR1 expression was determined using mAbs specific for ACKR1 (Thiriot et al., 2017), VE-Cadherin (Thermo Fisher Scientific) and CD31. Where indicated endothelial ACKR1 and chemokine MFI values were quantified within EC-junctions (isosurface created on $CD31^{high}$ or VE-Cadherin^{+} regions) or non-junctional regions (isosurface on $CD31^{dim}$/VE-Cadherin^{-} regions). All protein expression levels were quantified from 4-10 images per mouse and

expressed as MFI values per unit area of tissues stained with specific antibodies subtracted by MFI values per unit area of tissues stained with appropriate isotype controls. All images used for protein and mRNA (FISH) quantifications were captured with the 63x objective in the multiple track scanning mode every 0.43 μm at a resolution of 1024 × 512 μm corresponding to a voxel size of 0.099 × 0.099 × 0.43 μm in x × y × z, respectively. The fluorescence intensity line profiles were determined using ImageJ software (NIH).

Peritoneal inflammation

Peritonitis was induced by intraperitoneal (i.p.) injections of 300 ng TNF in WT mice or *Cxcl2*$^{-/-}$, *Ackr1*$^{-/-}$ or corresponding control chimeras, whereas non-inflamed control mice received 1 mL of PBS. Blocking anti-CXCL1, anti-CXCL2, anti-CXCR2 mAbs (R&D Systems) or corresponding isotype control mAbs (3 mg/kg, R&D Systems) were administered i.v. 10 min prior to TNF. 4 hr after TNF or PBS administration, mice were culled and subjected to peritoneal lavages using 5 mL PBS containing 5 mM EDTA (Sigma-Aldrich) and 0.25% BSA. The peritoneal exudates were stained for the leukocyte marker CD45, the neutrophil marker Ly6G (both Biolegend) and the monocyte and macrophage marker CD115 (Thermo Fisher Scientific) and total numbers of infiltrating neutrophils and monocytes per peritoneal cavity were determined by flow cytometry.

Flow cytometry

Where required, samples were treated with ACK buffer (150 mM NH_3Cl, 1 mM $KHCO_3$ and 1 mM EDTA) to lyse red blood cells. Subsequently, the samples were incubated with anti-CD16/-CD32 antibodies (Becton Dickinson) to block Fc-receptors and stained with primary fluorescently labeled antibodies of interest. CXCR2 surface levels on neutrophils were determined with an anti-CXCR2 mAb (Biolegend). The samples were analyzed on an LSR Fortessa flow cytometer (Becton Dickinson) and FlowJo software (TreeStar). Total neutrophil numbers in samples were determined using fluorescent counting beads (Thermo Fisher Scientific).

Confocal intravital microscopy (IVM)

The mode and dynamics of neutrophil migration through blood vessel walls was analyzed by confocal IVM, as described previously (Proebstl et al., 2012; Woodfin et al., 2011). In order to analyze neutrophil interactions with ECs and pericytes simultaneously *Lyz2-EGFP-ki*;*Acta2-RFPcherry-Tg* mice (exhibiting GFP$^+$ neutrophils and RFP$^+$ pericytes), were injected i.s. with an Alexa Fluor 647-labeled non-blocking anti-CD31 mAb (4 μg, clone 390, Thermo Fisher Scientific) to stain EC junctions (2 h). Alternatively, IVM was performed on bone marrow chimeric mice exhibiting *Lyz2-EGFP-ki* hematopoietic (GFP$^+$ neutrophils) and *Ackr1*$^{-/-}$ or WT non-hematopoietic cells, which received i.s. injections of an Alexa Fluor 555-anti-CD31 mAb (clone 390).

2 hr after i.s. administration of 300 ng TNF, 50 ng IL-1β or 400 μl PBS the mice were anaesthetized by i.p. administration of ketamine (100 mg/kg) and xylazine (10 mg/kg) and the cremaster muscles were exteriorized and pinned out flat over the optical window of a heated custom-built microscope stage. The animals were maintained at 37°C and the cremaster muscles were perfused with 37°C warm Tyrode's solution (Sigma-Aldrich) during the experiment. Blocking anti-CXCL1, anti-CXCL2 or isotype control mAbs (3 mg/kg) were injected i.v. 10 min before i.s. TNF administration for the analysis of intraluminal and TEM neutrophil responses as indicated. For the analysis of TEM and post-TEM neutrophil responses the mAbs (30 μg) were injected i.s. 100 min after TNF administration. Post-capillary venules with a diameter of 20-45 μm were recorded for 0.5-2 hr using an upright Leica SP5 confocal laser scanning microscope and a 20x water-dipping objective (NA 1.0). Serial z stack images were acquired every 30 or 60 s and assembled offline into 3D videos using Imaris software. The dimension of the recorded area was typically 300 × 130 × 35 μm and the resulting voxel size was 0.29 × 0.29 × 0.69 μm in x × y × z. Models of half vessel were generated to clearly visualize individual neutrophils migrating through the different compartments of venular walls. Neutrophil migration mode and dynamics (speed and displacement) were determined by manual tracking of individual neutrophils using Imaris software and migratory paths were graphically illustrated using the Chemotaxis and migration tool (IBIDI). Luminal neutrophils were defined as adherent when they remained stationary on the endothelium for at least 30 s and as intraluminally crawling when they exhibited a displacement of at least 2 cell diameters on the endothelium over the entire observation period. The numbers of adherent and crawling neutrophils were expressed as means determined at 4 time points per mouse. Neutrophil TEM was defined as an event where neutrophils fully migrated through EC junctions in a luminal-to-abluminal direction. Aborted TEM was classified as a response where luminal neutrophils extended protrusions through EC junctions (but did not exhibit complete TEM) and subsequently reverse migrated in an abluminal-to-luminal direction and finally disengaged from the EC junction and re-entered the vascular lumen. Reverse neutrophil TEM was a response where sub-EC neutrophils (after having fully breached the endothelium) migrated through an EC junction in an abluminal-to-luminal direction. TEM, aborted TEM and reverse TEM events were quantified over the entire IVM observation period of 2 h.

Neutrophil isolation

Murine neutrophils were isolated from bone marrow cells (harvested from tibiae and femora) or peripheral blood by negative magnetic cell sorting using the Neutrophil isolation kit (Miltenyi Biotec) according to the manufacturer's instructions. The purity of isolated neutrophils (CD45$^+$/Ly6G$^+$/CD115$^-$ expression profile) was consistently > 95% as determined by flow cytometry.

Ca^{2+} flux assay

Up to 10 × 10^6 isolated bone marrow neutrophils from WT C57BL/6 mice were suspended in 1 mL RPMI-1640 medium (Sigma-Aldrich) supplemented with 5% FCS, 2 mM L-glutamine, 1 g/L $NaHCO_3$, and 20 mM HEPES and incubated with 4 μM of the fluorescent Ca^{2+} indicator Fluo-4, 0.04% Pluronic F-127 and 1 mM Probenecid (all Thermo Fisher Scientific) for 45 min at 37°C. The neutrophils

were then washed and analyzed by flow cytometry at room temperature. Specifically, Fluo-4 fluorescence readings (excitation wavelength: 488 nm, 530/30 nm bandpass filter) were recorded for 30 s to establish a baseline and for 3 min after the addition of CXCL1 or CXCL2 (Preprotech). Results show baseline corrected Fluo-4 MFI or peak fluorescence values as determined using FlowJo software.

In vitro neutrophil adhesion assay

96 well plates were coated with 2.5 μg/mL ICAM-1 (R&D Systems) over night at 4°C and blocked with 10% low endotoxin BSA (Sigma-Aldrich) for 2 hr at room temperature. Isolated bone marrow neutrophils were added, and the plates were centrifuged at 20 g for 2 min and treated with CXCL1, CXCL2 or control medium for 15 min at 37°C. Non-adherent cells were then washed away with PBS containing 1 mM $CaCl_2$, 0.5 mM $MgCl_2$. Subsequently, adherent neutrophils were detached by TrypLE express cell detachment solution (Thermo Fisher Scientific) and quantified by flow cytometry. The results were expressed as the percentage of adherent neutrophils after chemokine addition, subtracted by the percentage of adherent neutrophils in the absence of chemokines.

Western Blot

Isolated neutrophils were lysed in 1x Laemmli Buffer, denatured at 95°C and subjected to standard Western Blot analysis using anti-pan-AKT and anti-phospho-AKT primary antibodies (Cell Signaling Technology) and a horseradish peroxidase-conjugated secondary antibody (Dako). Proteins were visualized by enhanced chemiluminescence acquired on X-ray film (Fuji Medical) and quantified by ImageJ software.

Transwell chemotaxis assay

Bone marrow neutrophils from *Lyz2-EGFP-ki* mice or mixed $Cxcl2^{-/-}$-$Cxcl2^{wt/wt}$ chimeric mice were seeded into top chambers of Transwell plates (3 μm pore diameter, Sigma-Aldrich) in PBS supplemented with 1 mM $CaCl_2$, 0.5 mM $MgCl_2$, 10 mM glucose, 10 mM HEPES (Sigma Aldrich) and 0.25% low endotoxin BSA. In some experiments, Transwell filters were coated with 0.5 μg/mL CXCL1 or CXCL2 in PBS or with PBS alone over night at 4°C and blocked with 10% low endotoxin BSA for 1 hr at room temperature before adding neutrophils. Where indicated 10 nM CXCL1 or CXCL2 were added to the top chamber with the neutrophils. 0-10 nM CXCL1, CXCL2 or leukotriene B_4 (LTB_4, Cayman Chemical) were added to the bottom chambers and the Transwell plates were incubated for 1 hr at 37°C. Neutrophils migrated into the bottom chambers were resuspended in PBS containing 5 mM EDTA and their absolute numbers were determined by flow cytometry. *GFP-*$Cxcl2^{wt/wt}$ and $Cxcl2^{-/-}$ neutrophils were identified by their $Ly6G^+/GFP^+$ or $Ly6G^+/GFP^-$ expression profile, respectively. Confocal microscopy was used to assess chemokine immobilization on Transwell filters by immunostaining and to analyze neutrophil morphology.

Fluorescence *in situ* hybridization (FISH)

Cremaster muscles were frozen and cut into 30 μm sections. *In situ* hybridization was carried out using the RNAscope fluorescent multiplex assay (Advanced Cell Diagnostics) according to the manufacturer's instruction with slight modifications. Briefly, after dehydration the sections were incubated with Pretreat 4 for 20 min at room temperature and hybridized with probes for *Pecam1, Acta2, Cxcl1 and Cxcl2* mRNAs for 2 hr at 40°C. To evaluate the assay, sections were hybridized with probes for *Ppib* (positive control) and *DapB* (negative control). The amplification steps were performed according to the RNAscope protocol. Where required, *in situ* hybridization procedure was directly followed by IF staining with an anti-α-SMA antibody over night at 4°C. Fluorescent mRNA spots and IF stainings were visualized by confocal microscopy. *Cxcl1* and *Cxcl2* mRNA copy numbers in microvascular walls were determined by automatic quantification of fluorescent spots within *Pecam1*- and *Acta2*-positive regions using Imaris software. Within this assay, as detailed in the manufacturer's guidelines, each mRNA molecule hybridized to a probe appears as single fluorescent spot. At least 6 vessel segments per mouse were analyzed.

Pericyte isolation and culture

Cremaster muscles from *Acta2-RFPcherry-Tg* mice were digested with 500 U/mL Collagenase II (Worthington) in PBS for 45 min at 37°C and 50 U/mL DNase I (Sigma-Aldrich) was added during the last 20 min. The resulting cell suspension was seeded onto tissue culture plates coated with gelatin and collagen I (Advanced BioMatrix) and cultured in low glucose Dulbecco's Modified Eagle's Medium (DMEM) supplemented with 10% FCS, 100 U/mL penicillin, 100 mg/mL streptomycin (all Thermo Fisher Scientific) and 100 pM pigment epithelium-derived factor (PEDF, Sigma-Aldrich). After ~21 days of culture, confluent cells were detached with 5 mM EDTA and cells exhibiting the unique venular pericyte signature (α-SMA^+PDGFR-β^+NG2^-) were isolated using the FACSCalibur cell sorter (Becton Dickinson). α-SMA^+ cells were identified by RFP expression. Pericytes showing > 90% purity were subjected to further analyses.

Real-time PCR

Total RNA was purified from isolated murine peripheral blood neutrophils or cultured lung ECs or cremaster muscle pericytes using the RNeasy micro kit (Quiagen) and reverse transcribed into cDNA with the iScript cDNA synthesis kit (Biorad). Quantitative real-time PCR was carried out using the iQ SYBR Green supermix (Biorad) according to the manufacturer's protocol, primers for *Cxcl1*, *Cxcl2* and *Gapdh* (Integrated DNA Technologies) and the 7900HT real-time PCR machine (Applied Biosystems). *Cxcl1* and *Cxcl2* mRNA levels were expressed in relation to *Gapdh*.

In vitro pericyte and neutrophil analysis

Cultured murine primary cremaster muscle pericytes were seeded onto gelatin- and collagen I-coated plates and activated with TNF in DMEM low glucose medium containing 10% FCS and 100 U/mL penicillin and 100 mg/mL streptomycin for 4 hr at 37°C. Alternatively, isolated bone marrow neutrophils were stimulated with 1 nM TNF in PBS containing 1 mM $CaCl_2$, 0.5 mM $MgCl_2$, 10 mM glucose, 10 mM HEPES and 0.25% low endotoxin BSA for 1 hr at 37°C. For some experiments, neutrophils were seeded on wells that were coated with 2.5 μg/mL CXCL1 or PBS over night at 4°C and blocked with 10% endotoxin low BSA for 1 hr at room temperature. Supernatants of pericyte and neutrophil cultures were taken at the end of the stimulation periods and the cells were lysed with 1% Triton X-100 in PBS containing HALT protease and phosphatase inhibitor (Thermo Fisher Scientific). Chemokine levels were determined by ELISA kits (R&D Systems, sensitivity: 2 pg/mL for CXCL1 and 1.5 pg/mL for CXCL2).

Generation of bone marrow chimeric mice

Mice exhibiting CXCL2-deficiency in the hematopoietic compartment and WT control chimeras were generated by transferring bone marrow cells from *Cxcl2*$^{-/-}$ or WT mice into *Lyz2-EGFP-ki* mice. Mixed *Cxcl2*$^{-/-}$-*Cxcl2*$^{wt/wt}$ chimeras were established by transferring a 1:1 mixture of bone marrow cells from *Cxcl2*$^{-/-}$ and *Lyz2-EGFP-ki* mice into *Lyz2-EGFP-ki* mice. *GFP-Cxcl2*$^{wt/wt}$ neutrophils in mixed chimeras were distinguished from *Cxcl2*$^{-/-}$ neutrophils based on their GFP expression. Mice exhibiting GFP^+ myeloid cells and *Ackr1*$^{-/-}$ or WT non-hematopoietic cells were generated by transferring bone marrow cells from *Lyz2-EGFP-ki* mice into *Ackr1*$^{-/-}$ or WT recipients. To generate the chimeras, recipient mice were lethally irradiated with 2 doses of 5 Gy given 4 hr apart. The following day 1.5×10^6 bone marrow cells from donor mice were injected i.v. into the irradiated mice. The chimeras were subjected to IVM analyses 4 weeks after bone marrow transplantation. Control experiments confirmed that *Cxcl2*$^{-/-}$ chimeras and mixed *Cxcl2*$^{-/-}$-*Cxcl2*$^{wt/wt}$ chimeras showed normal circulating neutrophil numbers compared to WT control chimeras (2442, 2509 and 2410 neutrophils/μl blood, respectively, $p > 0.5$, n = 11-20 mice per group). *Ackr1*$^{-/-}$ and WT chimeras expressed similar levels of CXCR2 on circulating neutrophils (MFI of 1100 and 975, respectively, $p > 0.5$, n = 3 mice per group), as determined 3 hr after i.s. injection of TNF.

Dorsal skin inflammation

TNF, LPS (Sigma-Aldrich, both 300 ng in 50 μl volumes) or PBS were injected intradermally into the dorsal skin of *Cxcl2*$^{-/-}$, *Ackr1*$^{-/-}$ or corresponding WT control chimeric mice. After 4 h, skin samples were dissected, frozen in liquid nitrogen and homogenized in homogenization buffer (600 mM NaCl, 0.5% hexadecyltrimethylammonium bromide buffer, 600 mM KH_2PO_4, 66 mM Na_2HPO_4) using a Precellys instrument (Bertin Technologies). Tissue debris was removed by centrifugation. The peroxidase activity in the supernatants was determined by adding the MPO substrate 3,3′,5,5′-tetramethylbenzidine (Invitrogen) and measuring the absorbance at 650 nm over 20 min using a Spectra MR photometer (Dynex technologies). The MPO activity (used as a readout for neutrophil infiltration) was expressed as the increase in optical density per min multiplied by 100.

In vivo rmCXCL2 and rmCXCL1 binding assay

RmCXCL2 or rmCXCL1 (both 0.5 μg) were injected i.s. into WT or *Ackr1*$^{-/-}$ mice together with an Alexa Fluor 488-anti-CD31 mAb (4 μg). After 2 h, the mice were culled, and the cremaster muscles were dissected, rinsed in cold PBS, fixed with 4% PFA, permeabilized and blocked with 0.5% Triton X-100 and 25% FCS in PBS and immunostained using antibodies specific for CXCL2 or CXCL1, MRP14 and VE-Cadherin or ACKR1. Subsequently, the tissues were analyzed by confocal microscopy. The biological activity of injected rmCXCL1 and rmCXCL2 was verified by intense neutrophil infiltration (not shown) and the binding capacity of rmCXCL1 was indicated via detection on extravascular cells (Figure S5E).

QUANTIFICATION AND STATISTICAL ANALYSES

Statistical analyses were performed using Prism software (GraphPad). The results are expressed as means ± SEM and the exact n numbers for each dataset is provided in the Figure legends. Comparisons between two groups were carried out using the paired or unpaired Student's t test or Fisher's exact test as appropriate. One-way ANOVA followed by Bonferroni post hoc test or two-way ANOVA with Holm Sidak's post hoc test were performed for multiple group comparisons. Differences between dose-response curves were evaluated with the extra-sum-of-squares F test. Statistical significance was accepted at $p < 0.05$.

Immunity, Volume *49*

Supplemental Information

Distinct Compartmentalization of the Chemokines CXCL1 and CXCL2 and the Atypical Receptor ACKR1 Determine Discrete Stages of Neutrophil Diapedesis

Tamara Girbl, Tchern Lenn, Lorena Perez, Loïc Rolas, Anna Barkaway, Aude Thiriot, Carlos del Fresno, Eleanor Lynam, Elin Hub, Marcus Thelen, Gerard Graham, Ronen Alon, David Sancho, Ulrich H. von Andrian, Mathieu-Benoit Voisin, Antal Rot, and Sussan Nourshargh

Supplemental Information

Supplemental Figures

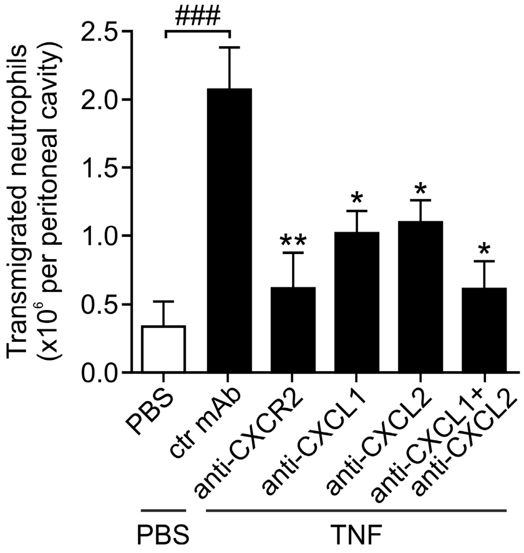

Figure S1 (related to Figure 1): TNF-induced neutrophil migration into the peritoneal cavity is dependent on both CXCL1 and CXCL2. Neutrophil extravasation in the peritoneal cavity of WT mice after intraperitoneal (i.p.) administration of PBS or TNF (300 ng, 4 h). Control (ctr) or blocking mAbs against CXCR2, CXCL1, CXCL2 or CXCL1 + CXCL2 were injected intravenously (i.v.) 10 min prior to TNF (n = 3 -14 mice per group, 9 independent experiments). Means ± SEM, *p<0.05, **p<0.01 as compared to TNF + ctr mAb treatment and ###p< 0.001 as indicated.

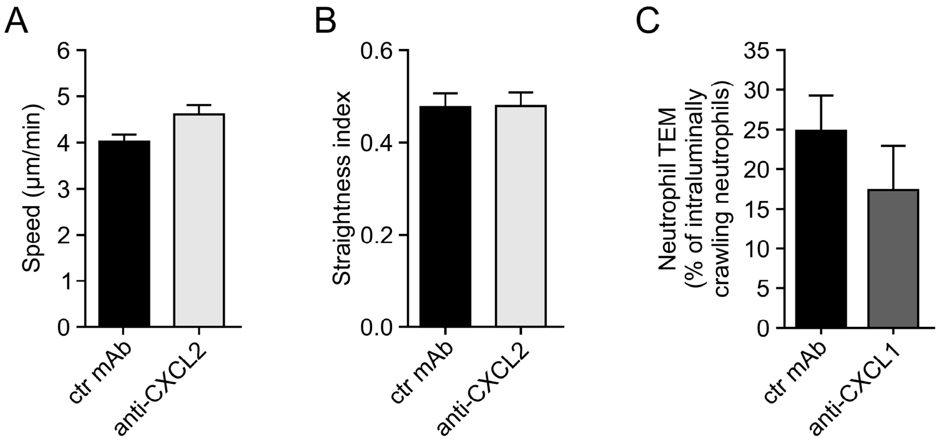

Figure S2 (related to Figure 2): Intraluminal neutrophil crawling dynamics and transendothelial cell migration (TEM) in TNF-stimulated cremaster muscles. (A-B) Intraluminal neutrophil crawling speed (A) and straightness (straightness index = displacement/length of migratory path, B) in TNF-stimulated cremaster muscles of *Lyz2-EGFP-ki;Acta2-RFPcherry-Tg* mice injected i.v. with ctr or blocking anti-CXCL2 mAbs (10 min prior to TNF) as determined by confocal IVM (n = 6 mice per group) from 12 independent experiments. (C) Percentages of intraluminally crawling neutrophils that exhibited TEM in TNF-treated cremaster muscles post treatment with i.s. ctr or anti-CXCL1 mAb (n = 5-10 mice per group) involving 15 independent experiments. Means ± SEM.

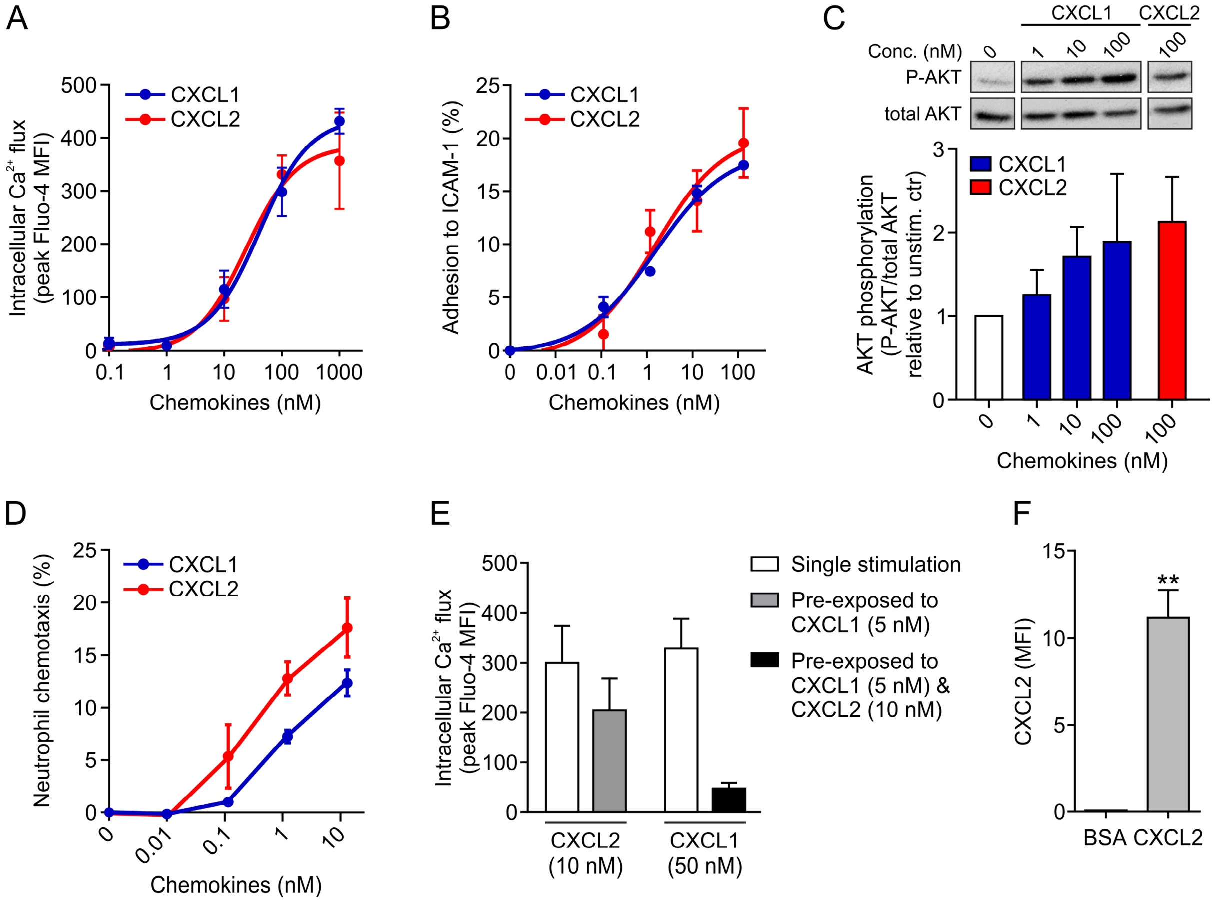

Figure S3 (related to Figure 4): CXCL1 and CXCL2 exhibit similar potencies in inducing neutrophil responses *in vitro*. (A) Intracellular Ca^{2+} flux in isolated bone marrow neutrophils loaded with the calcium indicator Fluo-4 and stimulated with CXCL1 or CXCL2. Peak Fluo-4 mean fluorescence intensities (MFIs) within 2 min after chemokine stimulation minus baseline MFIs, as determined by flow cytometry from 3 independent experiments are shown (n = 3). (B) Neutrophil adhesion to ICAM-1-coated plates as induced by CXCL1 or CXCL2 (n = 2) from 2 independent experiments. (C) Representative Western blot analysis of phospho-AKT (P-AKT) and total AKT and quantification of P-AKT in neutrophils stimulated with CXCL1 or CXCL2 (n = 6) from 4 independent experiments. (D) Neutrophil chemotaxis in response to CXCL1 or CXCL2 as determined by Transwell chemotaxis assays (n = 4) from 4 independent experiments. (E) Quantification of intracellular Ca^{2+} flux in neutrophils after single or serial stimulation with CXCL1 and CXCL2 as expressed as peak Fluo-4 MFIs (n = 4) from 4 independent experiments. (F) MFI of CXCL2 immunostaining on Transwell filters coated with BSA or CXCL2 (n = 3) involving 3 independent experiments. Means ± SEM, **p<0.01.

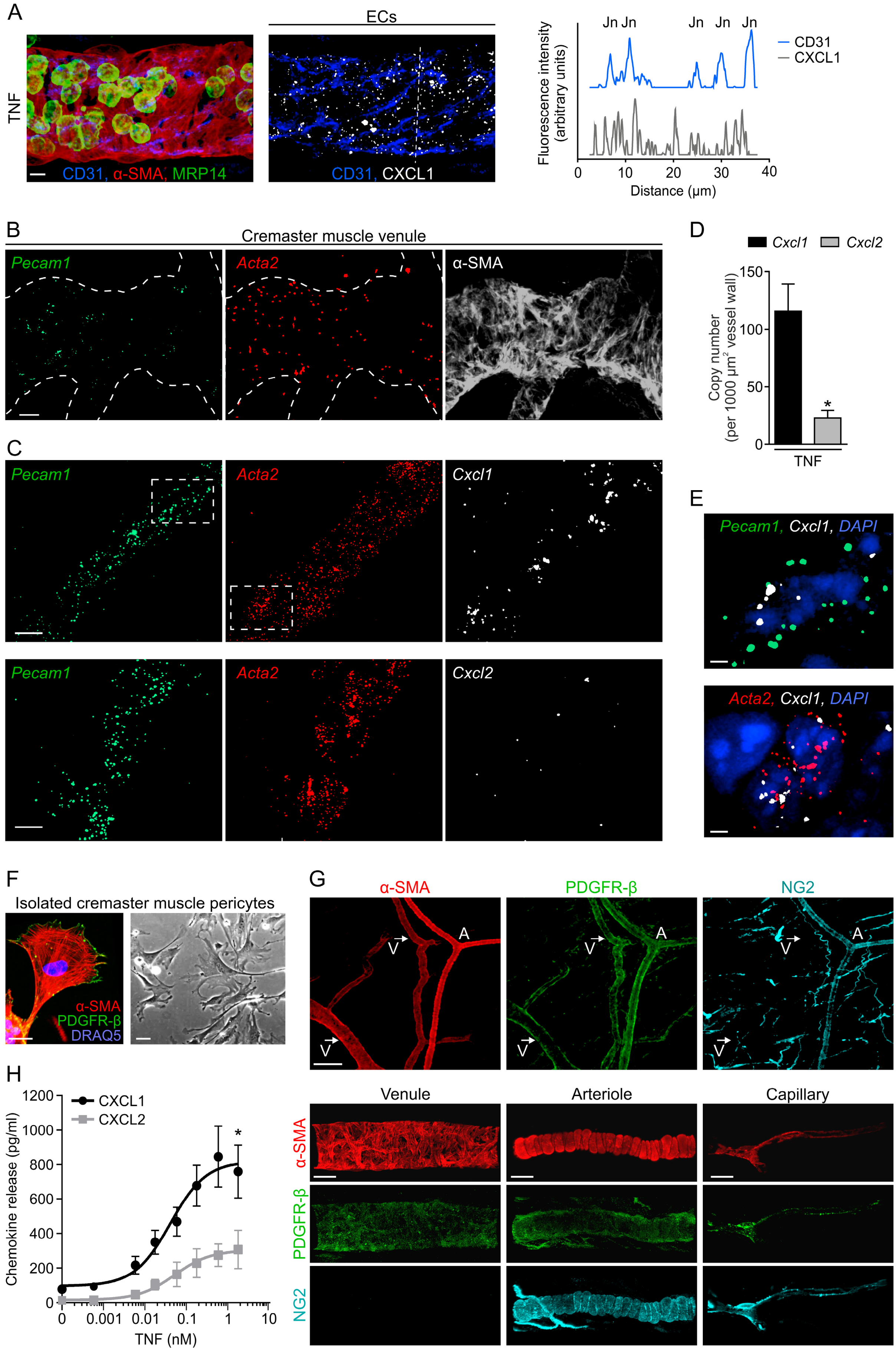

Figure S4 (related to Figure 5): Cremaster muscle ECs and pericytes are an abundant source of CXCL1. (A) TNF-stimulated cremaster muscles were immunostained for CXCL1,

CD31 (ECs), α-SMA (pericytes) and MRP14 (neutrophils) (left panel shows a representative confocal image). Representative confocal image illustrating CXCL1 staining within an EC isosurface mask (middle) and CD31 and CXCL1 immunofluorescence (IF) intensities along the dashed line cutting across 5 EC junctions (Jn, right) are shown. Images and line intensity graphs are representative of 4 independent experiments. Scale bar, 5 µm. (B-E) RNA fluorescent *in situ* hybridization (FISH) of TNF-stimulated cremaster muscles. (B) FISH was validated for the analysis of mRNAs in venular walls by robust detection of *Pecam1* and *Acta2* mRNAs (established markers for EC and pericytes, respectively), in venules concomitantly IF stained for α-SMA and analyzed by confocal microscopy. (C) Confocal images showing hybridization signals for *Pecam1*, *Acta2* and *Cxcl1* (top) or *Cxcl2* (bottom). Images in B and C are representative of 3 independent experiments. Scale bars, 10 µm. (D) Quantification of *Cxcl1* and *Cxcl2* mRNA copies in vascular walls (n = 3-6 mice per group) from 3 independent experiments. (E) Enlarged images of boxed regions in Panel C, showing *Cxcl1* and *Pecam1* in an EC (top) and *Cxcl1* and *Acta2* in a pericyte (bottom). Cell nuclei were stained with DAPI. Scale bars, 2 µm. (F-H) Isolation and analysis of murine cremaster muscle pericytes. (F) Isolated pericytes exhibited a venular pericyte expression profile (α-SMA$^+$PDGFR-β$^+$NG2$^-$) as determined by IF staining and confocal microscopy (left). Lack of expression of NG2 is not shown for clarity. The cells also displayed a stellate morphology that is typical for pericytes, as shown by phase contrast microscopy (right). Images are representative of 3 independent experiments. Scale bars, 30 µm. (G) The RFP$^+$PDGFR-β$^+$NG2$^-$ molecular signature uniquely distinguishes venular wall pericytes from arteriolar and capillary smooth muscle cells and pericytes *in vivo*, as shown by IF staining and confocal microscopy of cremaster muscles of WT mice. V, venule; A, arteriole. Scale bars, 100 µm (top) and 20 µm (bottom). The images are representative of 2 independent experiments. (H) CXCL1 and CXCL2 levels in the cell culture medium of isolated primary α-SMA$^+$PDGFR-β$^+$NG2$^-$ cremaster muscle pericytes stimulated with TNF for 4 h as quantified by ELISA (n = 3) from 3 independent experiments. Means ± SEM, *$p<0.05$ (maximal CXCL1 and CXCL2 release was compared in H).

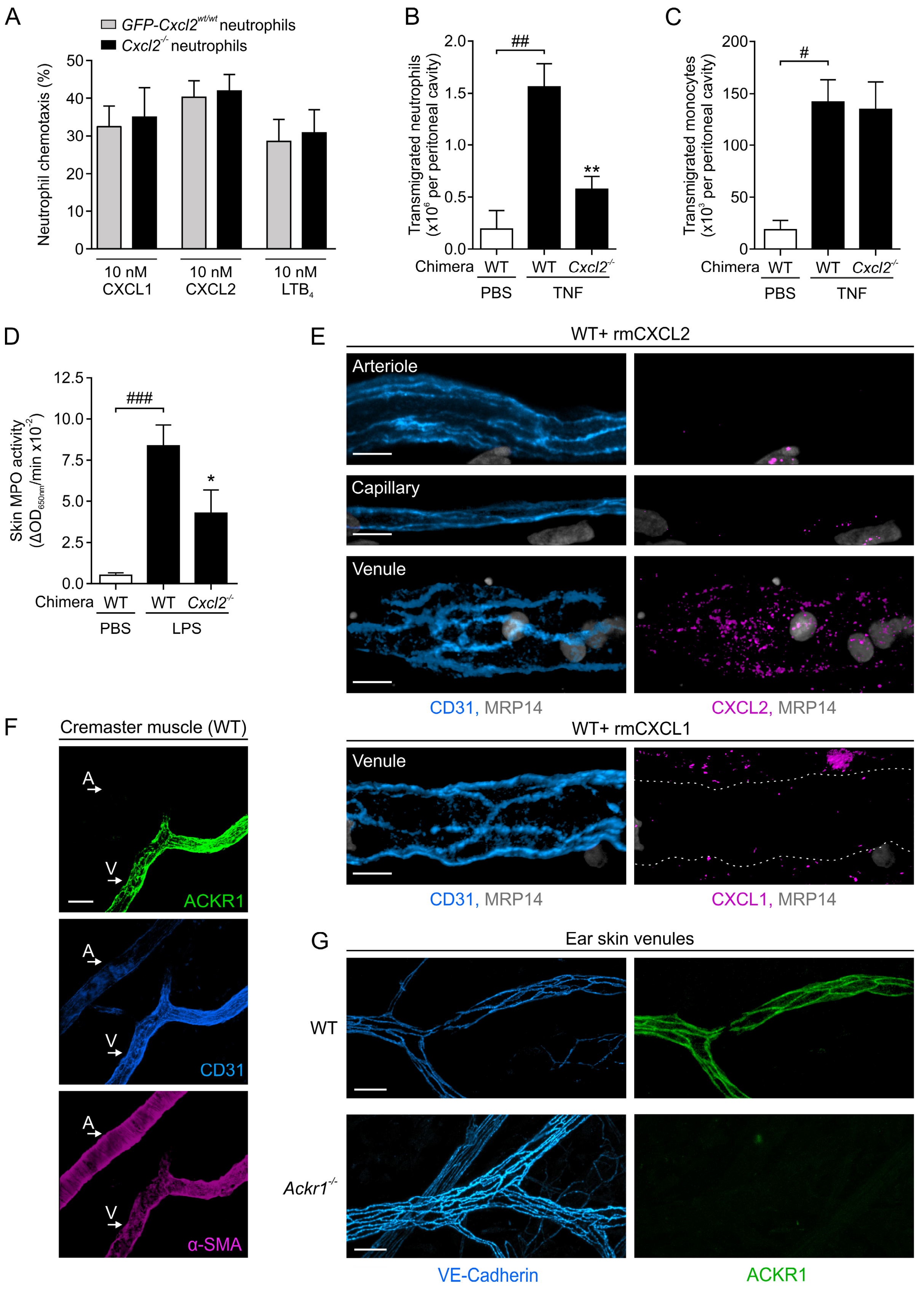

Figure S5 (related to Figure 6): The functional role of leukocyte CXCL2 in leukocyte extravasation and the expression profile of exogenous CXCL2 and CXCL1 and EC ACKR1. (A) Chemotaxis of *GFP-Cxcl2*$^{wt/wt}$ and *Cxcl2*$^{-/-}$ bone marrow neutrophils in response to CXCL1, CXCL2 or leukotriene B_4 (LTB_4) (10 nM each, 1 h) as determined by Transwell

assays (n = 3) from 3 independent experiments. (B-C) Neutrophil (B) and monocyte (C) extravasation into peritoneal cavities of WT or *Cxcl2*$^{-/-}$ chimeras injected i.p. with PBS or TNF injection (300 ng, 4 h, n = 4-10 mice per group, 4 independent experiments). (D) Neutrophil infiltration into dorsal skin sites of WT or *Cxcl2*$^{-/-}$ chimeric mice injected with PBS or lipopolysaccharide (LPS) (300 ng injected via the intradermal route, 4 h, n = 4-9 mice per group, 4 independent experiments), as quantified by myeloperoxidase (MPO) activity. (E) RmCXCL2 or rmCXCL1 was injected i.s. into WT mice and cremaster muscles were immunostained for CXCL2 or CXCL1, CD31 and MRP14. Confocal images of an arteriole, a capillary and venules are shown (representative of 8 independent experiments). Scale bars, 10 µm. Of note, levels of endogenously generated and EC-bound CXCL2 were below the detection limit of the present confocal microscopy platform. (F) Representative confocal images depicting a venule (V) and an arteriole (A) in a TNF-stimulated cremaster muscle IF stained for ACKR1, CD31 and α-SMA (representative of 3 independent experiments). Scale bar, 40 µm. (G) ACKR1 and VE-cadherin IF staining in venules of mouse ear skin in WT or *Ackr1*$^{-/-}$ mice (images are representative for 2 mice per group and 2 independent experiments). Scale bars, 30 µm. Means ± SEM, *p<0.05, **p<0.01 relative to WT chimeras and #p<0.05, ##p<0.01, ###<0.001 as indicated.

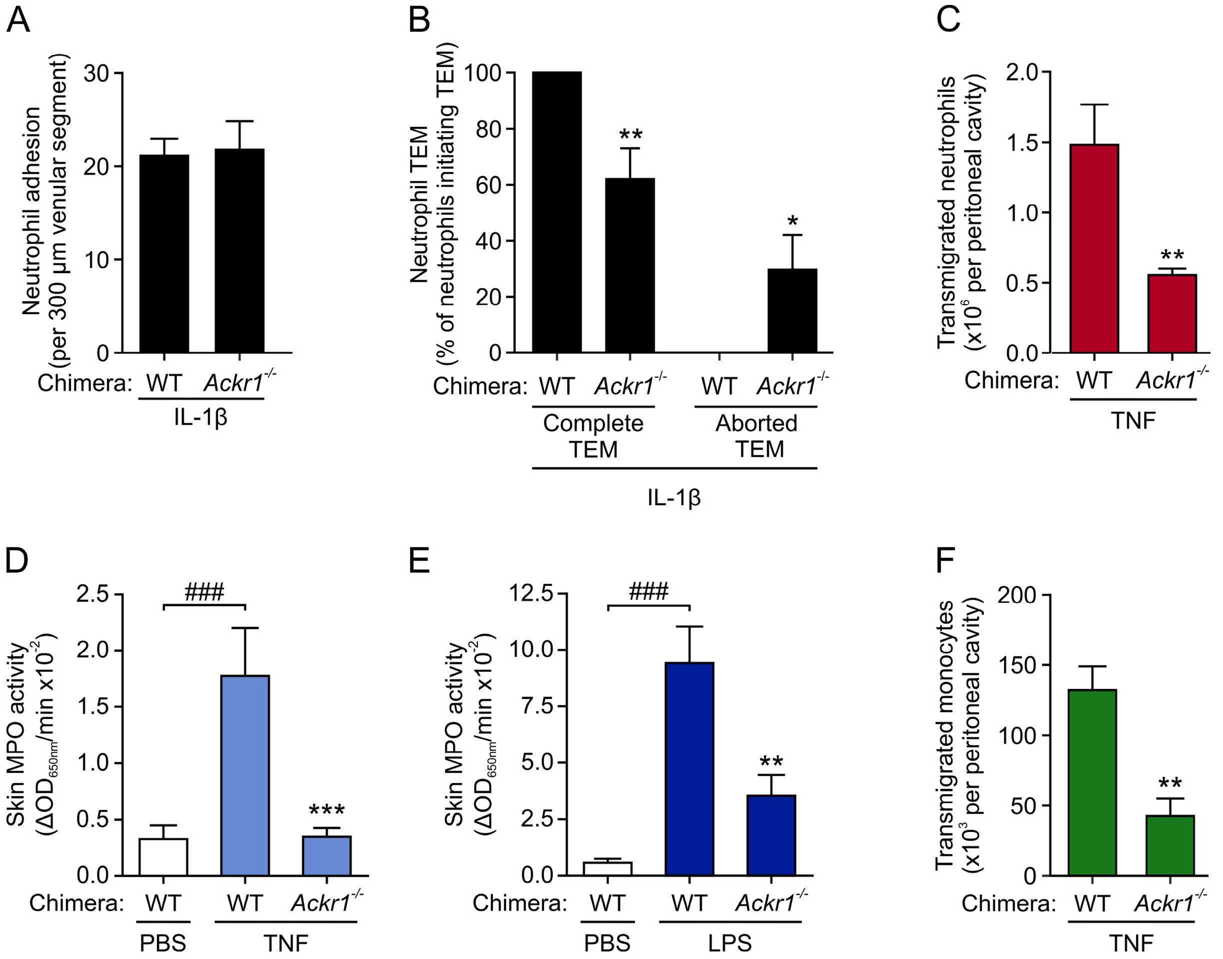

Figure S6 (related to Figure 7): EC ACKR1 facilitates neutrophil extravasation in multiple inflammatory models. (A-B) Neutrophil adhesion to ECs (A), complete TEM and aborted TEM (B) in cremaster muscle venules of WT or *Ackr1*$^{-/-}$ chimeric mice stimulated with IL-1β (50 ng, 4 h), as quantified by confocal IVM (n = 5-6 mice per group) from 11 independent experiments. (C) Neutrophil extravasation into the peritoneal cavity of WT and *Ackr1*$^{-/-}$ chimeric mice injected i.p. with TNF (300 ng, 4 h, n = 4-7 mice per group, 3 independent experiments). (D-E) Neutrophil infiltration into dorsal skin sites of WT or *Ackr1*$^{-/-}$ chimeric mice injected with PBS, TNF (D) or LPS (E) (both at 300 ng injected via the intradermal route, 4 h, n = 5-8 mice per group), as quantified by MPO activity from 4 independent experiments. (F) Monocyte extravasation in the peritoneal cavity of WT and *Ackr1*$^{-/-}$ chimeric mice injected i.p. with TNF (300 ng, 4 h, n = 4-7 mice per group, 3 independent experiments). Means ± SEM. *p<0.05, **p<0.01, ***p<0.001 as compared to WT chimeras and ### p< 0.001 as indicated.
